# De Novo Transcriptome Sequencing of Rough Lemon Leaves (*Citrus jambhiri* Lush.) in Response to *Plenodomus tracheiphilus* Infection

**DOI:** 10.3390/ijms22020882

**Published:** 2021-01-17

**Authors:** Riccardo Russo, Angelo Sicilia, Marco Caruso, Carmen Arlotta, Silvia Di Silvestro, Frederick G. Gmitter, Elisabetta Nicolosi, Angela Roberta Lo Piero

**Affiliations:** 1 CREA, Research Centre for Olive, Fruit and Citrus Crops, Corso Savoia 190, 95024 Acireale, Italy; riccardo.russo.1991@gmail.com (R.R.); marco.caruso@crea.gov.it (M.C.); arlottacarmen@gmail.com (C.A.); silvia.disilvestro@crea.gov.it (S.D.S.); 2 Department of Agriculture, Food and Environment, University of Catania, Via Santa Sofia 98, 95123 Catania, Italy; angelo.sicilia@unict.it (A.S.); enicolo@unict.it (E.N.); 3 Citrus Research and Education Center, Institute of Food and Agricultural Sciences, University of Florida, Lake Alfred, FL 33809, USA; fgmitter@ufl.edu

**Keywords:** *Plenodomus tracheiphilus*, *Citrus jambhiri*, rough lemon, mal secco, RNAseq, de novo assembly, SAR

## Abstract

Mal secco is one of the most severe diseases of citrus, caused by the necrotrophic fungus *Plenodomus tracheiphilus*. With the main aim of identifying candidate genes involved in the response of citrus plants to “Mal secco”, we performed a de novo transcriptome analysis of rough lemon seedlings subjected to inoculation of *P. tracheiphilus*. The analysis of differential expressed genes (DEGs) highlighted a sharp response triggered by the pathogen as a total of 4986 significant DEGs (2865 genes up-regulated and 2121 down-regulated) have been revealed. The analysis of the most significantly enriched KEGG pathways indicated that a crucial role is played by genes involved in “Plant hormone signal transduction”, “Phenylpropanoid biosynthesis”, and “Carbon metabolism”. The main findings of this work are that under fungus challenge, the rough lemon genes involved both in the light harvesting and the photosynthetic electron flow were significantly down-regulated, thus probably inducing a shortage of energy for cellular functions. Moreover, the systemic acquired resistance (SAR) was activated through the induced salicylic acid cascade. Interestingly, RPM1 interacting protein 4, an essential positive regulator of plant defense, and BIR2, which is a negative regulator of basal level of immunity, have been identified thus representing useful targets for molecular breeding.

## 1. Introduction

Citrus, one of the most important fruit crops in the world, is sensitive to many environmental stresses of both abiotic and biotic nature, often leading to poor tree growth and reductions in fruit yield and quality [1]. “Mal secco” disease (MSD) is a severe vascular disease of citrus caused by the mitosporic fungus *Plenodomus tracheiphilus* (Petri) Gruyter, Aveskamp and Verkley (syn. *Phoma tracheiphila* (Petri) Kantschaveli and Gikashvili). It appeared in the second half of 19th century (1894) in Chios and Poros, two Greek Aegean islands, from which it derived its first name (“Poros’s disease”). In Italy, MSD was first reported in 1918 in the district of Messina (eastern Sicily), probably following the introduction of infected plants from Greece [2]. The current geographical distribution of MSD comprises the east coast of the Black Sea (Georgia) and mainly all citrus-growing countries of the Mediterranean Basin, except for Morocco and Portugal [3].

The MSD pathogen infects mainly lemon (*Citrus limon* (L.) Burm. F.) [4]. Citron (*C. medica* L.) and other citrus species and hybrids having citron or lemon as parent, such as lime (*C. aurantifolia* Christ.), bergamot (*C. bergamia* Risso), Volkamer lemon (*C. volkameriana* Ten. et Pasq.), Alemow (*C. macrophylla* Wester), and rough lemon (*C. jambhiri* Lush) are also particularly susceptible to the disease [4,5]. Rough lemon is counted among the most “mal secco” susceptible species [6]. *C. jambhiri* Lush is native to northeastern India and is a mandarin × citron F1 natural hybrid [7]. Due to fruit typology, as the name implies, characterized by a very coarse exterior, it is unsuitable as a scion cultivar but it has been widely used in many countries as a rootstock [8].

The distinct symptomatology of the disease, characterized by desiccation of twigs, branches, or the whole plant, suggested its extant name “mal secco” meaning “dry disease” [9,10], a denomination ever since adopted internationally [5]. The first symptoms of the disease usually appear in spring on the leaves of the uppermost shoots, which display a slight discoloration of the primary and the secondary veins [11,12]. The leaves then turn yellow or sometimes brown and fall. Newly infected shoots show a yellow or pink-salmon to reddish discoloration of the wood, which occurs also in the wood of the main and secondary branches, as well as in the trunk, where the pathogen is advancing. A progressive basipetal desiccation of shoots, branches, and trunk follows and, finally, the whole plant may die [13]. Glycoproteins of 93 KDa and 60 KDa (called Pt60) belonging to the malseccin complex have been isolated from culture filtrates and host plants infected by *P. tracheiphilus* [14,15,16]. Both were able to reproduce all the symptoms of the disease when injected into different plants [16]. The toxic effects of the malseccin complex on citrus leaves are clearly visible only under illuminated conditions, suggesting that light plays a role in the toxin activity. In light conditions, the induction and formation of reactive oxygen species (ROS) can damage cellular structures as ROS induce lipid membrane peroxidation leading to the loss of membrane integrity, electrolyte leakage, and cell death. Oxidative stress in plant pathology has been a general subject of investigation and its ability to drive the metabolism of both host and pathogen during their interaction has been demonstrated [17]. It has been shown that the synchronous presence of hydrolytic enzymes, toxic compounds, oxidative stress inducers, and membrane transporters in the fungus, and the differential ability to modulate the lipoperoxidative pathway in the host can play a central function in *P. tracheiphilus* infection of *C. limon* [18].

The knowledge at the molecular level of the mechanisms that occur in plant–pathogen interaction, not only in tolerant but also in susceptible interactions, is the basis for the development of innovative tools for phytosanitary control and that may lead to eco-sustainable interventions to minimize or replace the massive use of agro-pharmaceuticals. Gene expression profiling by RNA-Seq provides an unprecedented high-resolution view of the global transcriptional landscape. A primary objective of many gene expression experiments is to detect transcripts showing differential expression across various conditions. In this context, next-generation high-throughput sequencing techniques have become an increasingly useful tool for exploring whole plant genomes, providing a means for analyzing plant molecular regulatory mechanisms in specific abiotic and biotic stress conditions. The identification of candidate genes is a prerequisite for the application of new genome editing techniques by which targeted genetic modifications can lead to the introduction of precise changes directly into the genome of commercial varieties, offering an alternative to traditional methods of genetic improvement [19,20,21]. Different authors in the last years conducted transcriptomic analysis to better understand *Citrus* plants response to biotic stress caused by pathogens [22,23,24,25,26,27]. Specifically, a study evaluated the transcriptional reprogramming of both rough lemon and sweet orange leaf tissue during the asymptomatic stage of infection caused by *Candidatus* Liberibacter asiaticus. Functional analysis of the differentially expressed genes (DEGs) indicated that genes involved in the mitogen activated protein kinase (MAPK) signaling pathway involving WRKY transcription factors were highly upregulated in rough lemon. Among the most biologically relevant transcripts in the gene set enrichment analysis were those related to several functional categories suggesting that DEGs with different functions were subjected to reprogramming. Therefore, using global transcriptome analysis approach, both a wide range of candidate genes and information that could be useful for genetic engineering to control Huanglongbing disease have been identified [25]. Considering the impact of mal secco in the Mediterranean citrus industry, the aim of this work was to unravel the transcriptomic reprogramming of a highly susceptible citrus species subjected to *P. tracheiphilus* infection by applying a de novo sequencing and assembly RNAseq approach. This is the first report concerning the transcriptome analysis of a susceptible Citrus species challenged by the causal agent of “mal secco” disease.

## 2. Results

### 2.1. Effect of Plenodomus tracheiphilus Infection on Citrus jambhiri Phenotype and Fungus Detection

The effectiveness of fungal inoculation was evaluated by both visual inspection of inoculated leaves and by detection of fungus genome by Taqman real time PCR. As shown in Figure S1A, the typical symptoms consisting of the midrib and main vein chlorosis were detected 15 days after inoculation. All the inoculated plants were chlorotic on the adaxial leaf surface (Figure S1B); that chlorosis symptom is different from the aforementioned vein chlorosis and more specifically indicates that a pathogen-induced micronutrient deficiency has occurred. As expected, the untreated plants appeared healthy (Figure S1B). As described in [28], quantitative detection of *P. tracheiphilus* was performed by real-time PCR assay. The fungus was detected in inoculated rough lemon plants, whereas no fluorescence emission was detected in the case of DNA extracted from healthy samples as well as from negative control (NTC, inoculated with water) (Table S1). The standard curve for fungal DNA quantification gave a coefficient of determination R^2^ = 0.98 (data not shown).

### 2.2. Transcript Assembly and Annotation

In this work, a comprehensive identification of the transcriptional response of rough lemon (*Citrus jambhiri* Lush) to *P. tracheiphilus* infection was carried out by RNAseq approach (see the experimental design in the “Material and Methods” section). The quality of RNA samples has been assessed before libraries preparation by RIN measurement. The mean RIN value was 8.2 (Table 1) indicating that very low level of RNA degradation occurred and that it was suitable for further downstream analysis.

**Figure 1:**
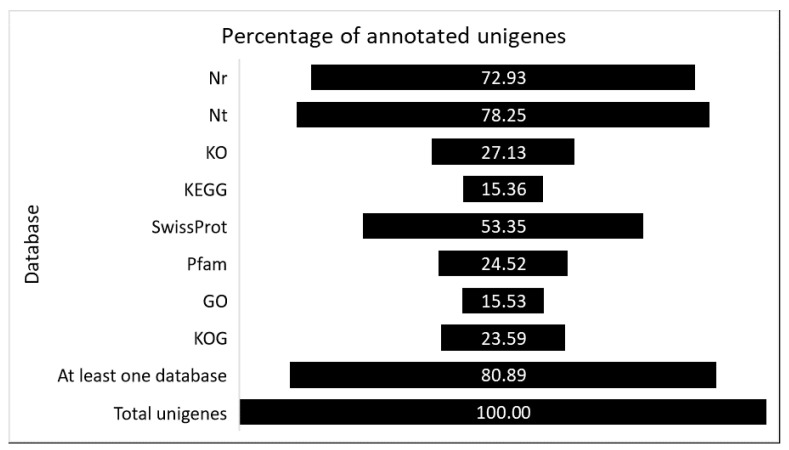
The percentage of successful annotated genes in several databases.

After library construction and sequencing, reads containing adapters or reads of low quality were removed by filtering the raw reads, so that the downstream analyses are based on a total of 228 million clean reads with an average of ~38 million reads (~11.4 G) per sample, the average percentage of Q30 and GC being 92.8% and 44.2%, respectively. De novo assembly of clean reads resulted in 115,100 transcripts and 77,631 unigenes with N50 length of 2372 and 2060, respectively (Table 1), indicating that a good coverage of the transcriptome had been achieved. The assembly consistency was evaluated by mapping back the filtered unique reads to the final assembled leaf transcriptome and the average read mapping rate using the alignment software Bowtie2 was 83.40%. Both transcript and unigene length distributions are reported in Figure S2. These data showed that the throughput and sequencing quality were high enough to warrant further analysis. To achieve comprehensive gene functional annotation, all assembled unigenes were blasted against public databases, including National Center for Biotechnology Information (NCBI), Protein family (Pfam), Clusters of Orthologous Groups of proteins (KOG/COG), SwissProt, Ortholog database (KO), and Gene Ontology (GO) (Figure 1). The 80.89% of the obtained total unigenes were annotated in at least one searched database. Among them, 72.93% and 78.25% assembled unigenes showed identity with sequences in the Nr and Nt databases, respectively. The percentage of assembled unigenes homologous to sequences in KO, KEGG, Swiss-Prot, Pfam, GO, and KOG databases were 27.13%, 15.36%, 53.35%, 24.52%, 15.53%, and 23.59%, respectively (Figure 1).

### 2.3. Identification of Differentially Expressed Genes (DEGs)

**Figure 2:**
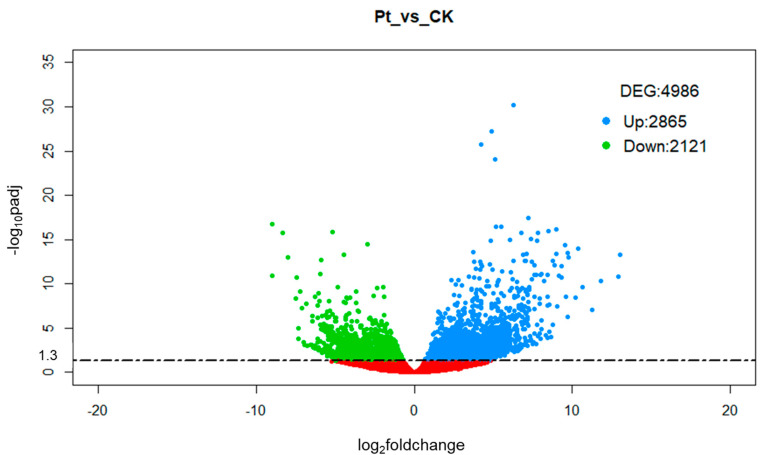
Volcano plot showing the DEGs of *Pt* vs. CK comparison. The up-regulated genes with statistically significance are represented by blue dots, the green dots represent the down-regulated genes and the red dots are DEGs with -log_10_padj < 1.3, adopting log_2_FoldChange threshold of 0.58 (1.5 fold change). The X-axis is the gene expression change, and the Y-axis is the *p* value adjusted after normalization.

The unigenes whose expression level changed upon pathogen infection were identified as differentially expressed genes (DEGs) and they were used to characterize the transcriptomic response of *C. jambhiri* to fungal attack. A total of 4986 differentially expressed genes were identified from the comparison *Pt* vs. CK (*P. tracheiphilus* sample set versus control sample set), of which 2865 were up-regulated and 2121 were down-regulated (Figure 2). Validation of expression levels for ten selected DEG candidates was carried out by quantitative real-time PCR (qRT-PCR). The results show high congruence between RNA-Seq results and qRT-PCR (coefficient of determination R^2^ = 0.92) indicating the reliability of RNA-Seq quantification of gene expression (Figure S3). Therefore, the selected genes could also constitute useful markers of pathogen infection in rough lemon.

### 2.4. Functional Classification of DEGs

**Figure 3:**
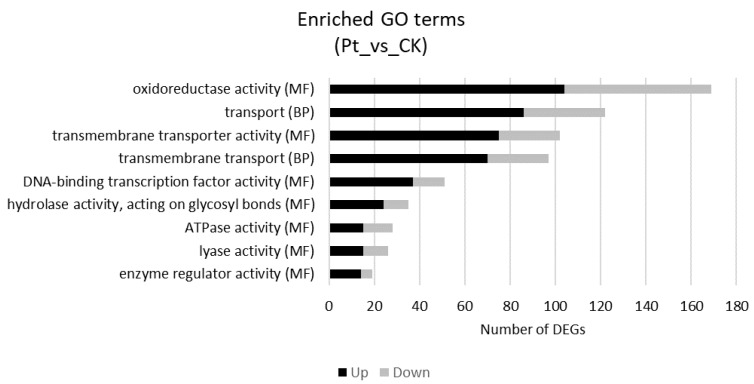
Gene Ontology (GO) enrichment analysis for the DEGs in *Citrus jambhiri* (*Pt* vs. CK comparison). The X-axis indicates the numbers related to the total number of GO terms, and the Y-axis indicates the subcategories. BP, biological processes; MF, molecular functions.

Gene Ontology (GO) terms, Clusters of Orthologous Groups of proteins (KOG) classification and Kyoto Encyclopedia of Genes and Genomes (KEGG) pathway functional enrichments were performed to identify possible biological processes or pathways involved in the response of plant to pathogen. Considering the GO enrichment, “oxidoreductase activity” (GO:0016491) (104 up-regulated and 65 down-regulated), “transmembrane transporter activity” (GO:0022857) (75 up-regulated and 27 down-regulated) and “DNA-binding transcription factor activity” (GO:0003700) (37 up-regulated and 14 down-regulated) are the three most enriched terms in Molecular Function (MF) category, while “transport” (GO:0006810) (86 up-regulated and 36 down-regulated) and “transmembrane transport” (GO:0055085) (70 up-regulated and 27 down-regulated) are the two most enriched terms in Biological Process (BP) category (Figure 3).

To predict and classify possible functions, all the 77,631 unigenes were aligned to the KOG database and were assigned to the KOG categories (Figure S4). Among the KOG categories, the cluster for “General function prediction only” (15.8%) represented the largest group, followed by “Posttranslational modification, protein turnover, chaperones” (12.9%) and “Signal transduction mechanisms” (9.1%). “Translation, ribosomal structure and biogenesis” (7.3%) and “RNA processing and modification” (6.8%) were the largest next categories (Figure S4).

**Figure 4:**
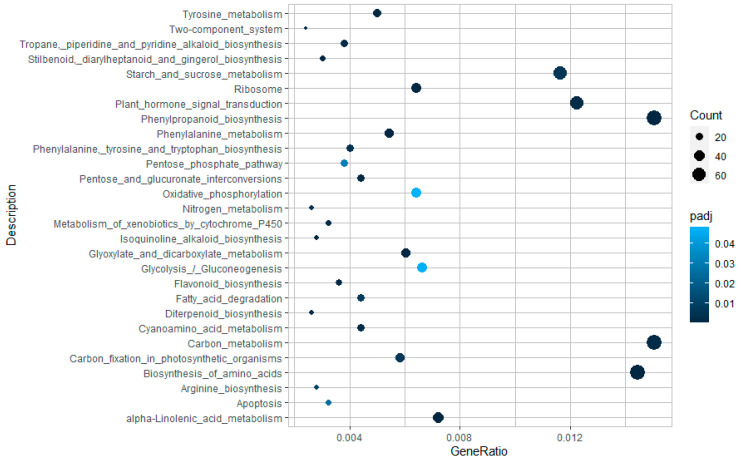
Distribution of Kyoto Encyclopedia of Genes and Genomes (KEGG) pathways for differential expressed genes (DEGs) in the *Pt* vs. CK sample set.

The main KEGG pathway terms were in the “Carbon metabolism” and “Phenylpropanoid biosynthesis” categories, followed by and “Biosynthesis of amino acids” indicating that a deep cellular rearrangement occurred in presence of the fungus (Figure 4). The reprogramming activity of the metabolic pathways is supported by the involvement of other important pathways such as “Plant hormone signal transduction” and “Starch and sucrose metabolism”. The strong involvement of “Plant hormone” category in the response to pathogen is also indicated by the presence of different pathways involved in amino acid biosynthesis and metabolism such as “Tyrosine metabolism”, “Phenylalanine metabolism”, “Phenylalanine, tyrosine and tryptophan biosynthesis”, and “Arginine biosynthesis”, known to be precursors of plant hormones (Figure 4).

**Figure 5:**
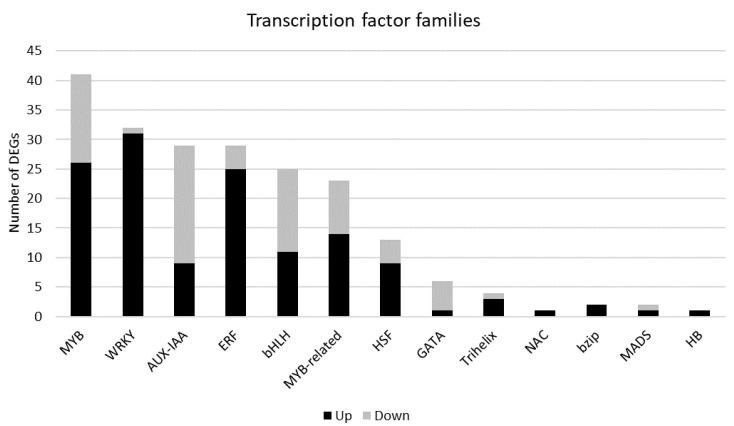
Distribution of rough lemon transcription factors responsive to *Plenodomus tracheiphilus* infection. Each bar represents the number of DEGs belonging to a transcription factor family.

Because of their fundamental involvement of “Plant hormone” (Table 2, Figure S5), “Transcription factors” (Figure 5) and “Defense and pathogenesis” related genes (Table 3) in host–pathogen interaction, we have analyzed them further. The following description of DEGs belonging to the above-mentioned pathways was carried out considering a log_2_foldchange threshold of ±2.32 (corresponding to a fold change = ±5). In the following tables, the coding sequence of each clusters were identified as orthologs of *A. thaliana* genes (http://plantgdb.org/prj/GenomeBrowser, accessed on 23 November 2020). Congruously, tables report clusters whose % of identity was higher than 50 and the e value < 0.05.

#### 2.4.1. Plant Hormone Related Genes

A significant deviation in the expression of genes involved in “Plant hormone” category was observed between the infected and control samples (Table 2, Figure S5). Considering auxin, known to be required for plant growth, the gene encoding one of the main biosynthetic enzymes, such as flavin-binding monooxygenase family protein YUC6 [29] was downregulated as well as the transmembrane amino acid transporter protein (AUX1) and three auxin-responsive IAA proteins (IAA32, IAA7, and IAA3) indicating that auxin biosynthesis and signaling are impaired in the inoculated plants. However, auxin-responsive transcription factors have been found up regulated suggesting that several pathways might be differently regulated. In this study, transcripts encoding several isoforms of the 1-amino-cyclopropane-1-carboxylate synthase, involved in the ethylene biosynthesis, were up-regulated. Moreover, many genes belonging to the ethylene signal transduction pathway and acting downstream of ethylene (signal transduction histidine kinase, hybrid-type, ethylene sensor (ETR2), mitogen-activated protein kinase 1 (MPK1), EIN3-binding F box protein 1 (EBF1/2), ethylene insensitive 3 family protein (EIN3), and ethylene response factor 1 (ERF1/2) were up-regulated (Table 2, Figure S5), clearly indicating an activation of ethylene signaling which might lead to the inhibition of plant growth and changes in a plant’s life cycle. Salicylic acid (SA) is synthesized via the shikimic acid pathway, with chorismic acid serving as an important precursor that can be converted to SA via two distinct branches. In one branch, chorismic acid is converted to SA via phenylalanine and cinnamic acid intermediates by the key enzyme phenylalanine ammonia lyase (PAL). In the other branch, chorismic acid is converted to SA via isochorismic acid by the enzyme isochorismate synthase (ICS1/SID2) [30]. Among the up-regulated transcripts, phenylalanine ammonia-lyase and 4-coumarate-CoA both implicated in one branch of salicylic acid biosynthesis have been found induced in the *Pt* vs. CK comparison. Moreover, genes encoding ICS1 were not among the DEGs suggesting that the main route for salicylic acid biosynthesis under biotic stress in rough lemon is that starting by phenylalanine and catalyzed by PAL.

#### 2.4.2. Transcription Factors

Reprogramming of gene expression upon *P. tracheiphilus* infection is regulated by many transcription factors. In Figure 5 the most represented transcription factor (TF) families in terms of number of DEGs are reported. The results showed that 41 DEGs belong to MYB family (26 up-regulated and 15 down-regulated), 29 to both auxin responsive protein (AUX/IAA) and ethylene-responsive transcription factor (ERF) families, these latter already cited above (*“Plant hormone related genes”* section) indicating that they play a key role in regulating the transcriptional response induced by the pathogenic fungal infection (Figure 5). In addition, 32 genes encoding WRKY transcription factors were among the DEGs, most of which were over-expressed (31 up-regulated and 1 down-regulated). Due to their involvement in plant response to pathogenic fungi infection [31,32,33,34,35] the analysis of their role are included in the following paragraph (Table 3).

#### 2.4.3. Defense and Pathogenesis Related Genes

In Table 3 differentially expressed genes involved in defense mechanisms and pathogenesis are summarized to provide a complete picture of the rough lemon response to pathogen attack. A plethora of genes encoding calmodulin-like protein, calcium-dependent protein kinase, mitogen-activated protein kinase 3, mitogen-activated protein kinase kinase kinase 15, mitogen-activated protein kinase kinase kinase 17, and GTPase activators were up-regulated in the *Pt* vs. CK sample set. These results clearly indicate that fungal infection triggers a wide reprogramming of the cellular signal transduction. Among the DEGs, several leucine rich repeat (LRR) domains, which might have a role as plant resistance (R) genes (IOS1, EIX2, and LECRK3) were up-regulated in the inoculated plants. However, the up-regulation of BIR2, which is negative regulator of basal level of immunity (namely PTI, pathogen-associated molecular patterns triggered immunity) strongly suggests that plant defense is already impaired at this first level [36]. Nevertheless, some of R genes are also known to activate prolonged resistance by inducing phytohormones and pathogenicity related genes (PR genes) that collectively give rise to broad spectrum systemic acquired resistance (SAR) against future infections [37]. Indeed, the members of the pathogenesis-related protein 1 (PR-1) family, which are among the most abundantly produced proteins in plants on pathogen attack, were up-regulated in rough lemon infected plants (Table 3). Concomitantly, genes encoding the positive regulator protein NPR1, which is involved in the induction of defense gene and PR-1 gene expression, and the TGA transcription factor that NPR1 interacts with in the nucleus, were up-regulated in the inoculated plants. These findings suggest that systemic acquired resistance (SAR) mechanism occurred in the rough lemon interaction with the pathogen, probably giving rise to broad-spectrum systemic protection against future infections. According to these results, another signal component of the SAR pathway BAD1, functioning upstream of NPR1 to regulate defense responses, was found to be induced by the pathogen in the *Pt* vs. CK comparison (Table 3). Finally, transcript encoding CERK1 Lysin motif (LysM) receptor kinase that functions as a cell surface receptor in chitin elicitor signaling involved in the resistance to pathogenic fungi [38] was up-regulated in the infected plants (Table 3). It probably acts by sensing microbe-associated molecular patterns (MAMP) and pathogen-associated molecular patterns (PAMP) as a component of the PTI. Finally, RPM1 interacting protein 4 is an essential regulator of plant defense, which plays a central role in resistance in case of infection; it acts in association with avirulence proteins with which it triggers a defense system including the hypersensitive response (HR) limiting the spread of disease. Interestingly, this transcript was found down-regulated in the inoculated plant (Table 3) suggesting that it might have a role in susceptibility of rough lemon which is not able to avoid the pathogen circulation inside the plant. Transcriptional regulation of defense related genes is crucial for defeating pathogens. The involvement of chitin elicitation that is suggested by the up-regulation of CERK1 appears to play a significant role in plant defense to fungal pathogens through the activity of transcription factors belonging to WRKY family [31]. Different genes encoding for WRKY DNA-binding protein were overexpressed in *C. jambhiri* infected plants. In detail, we observed the up-regulation of *WRKY14*, *WRKY23*, *WRKY49*, *WRKY72*, *WRKY75*, and *WRKY71*. Moreover, *WRKY4*, that is reported to have a positive role in resistance to necrotrophic pathogens [34], *WRKY51*, acts as positive regulator of salicylic acid (SA)-mediated signaling [33], *WRKY40*, *WRKY18,* and *WRKY70* specifically that responds to chitin [31] were also induced by *P. tracheiphilus* attack (Table 3). Finally, in response to pathogen infection, the induction of the calcium-dependent respiratory burst oxidase homologues (RBOHB, RBOHC, RBOHD, and RBOHF), which represent the major sources of ROS production in plants induced by pathogen infection, was observed in inoculated rough lemon plants [39].

#### 2.4.4. Main Processes or Pathways Affected by *P. tracheiphilus* Infection

**Figure 6:**
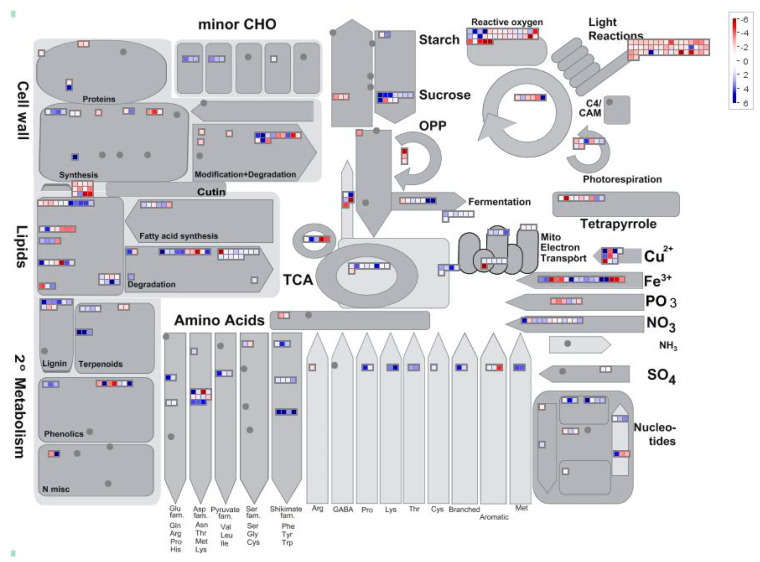
MapMan analysis of differentially expressed genes in *C. jambhiri* affected by *P. tracheiphilus.* Red dots represent up-regulated genes, blue dots represent down-regulated genes in the *Pt* vs. CK comparison.

In order to have a comprehensive view of the metabolic changes occurring in rough lemon infected by *P. tracheiphilus*, all the 4986 significant DEGs were mapped to the MapMan 3.6.0RC1 pathways, and the metabolism overview is shown in the Figure 6. Overall, the analysis indicates that the pathways which are more specifically involved in the response to *P. tracheiphilus* infection are “Reactive oxygen” (both up- and down- regulated genes), “Light reaction” (mostly down-regulated genes), “Nutrient homeostasis” (both up- and down- regulated genes), “Carbohydrate metabolism” (up-regulated genes), all of these will be singularly analyzed (Table 4).

##### Reactive Oxygen

Table 4 reports the DEGs related to “reactive oxygen” category. Two main gene sets were found to be strongly up-regulated in the *Pt* vs. CK comparison: Genes involved in the oxidoreductase activity and glutathione transferases. In particular, genes encoding copper/zinc superoxide dismutase, ascorbate peroxidase were induced by pathogen to overcome the damage induced by ROSs that play a central role during plant–necrotrophic fungus interactions through the stimulation of the plant’s defense responses [40]. The gene encoding allene oxide synthase, involved in the pathway of oxylipin biosynthesis starting from unsaturated fatty acids was found strongly up-regulated. Their chemical nature renders unsaturated fatty acids intrinsic antioxidants; that is, they can directly react with ROS and thus consume them. Their oxidation gives rise to various oxylipins that, in turn, modulates ROS levels and signaling [41]. Transcript of aldehyde dehydrogenase 3H1 involved in oxidative stress tolerance by detoxifying reactive aldehydes derived from lipid peroxidation was also found up-regulated in diseased rough lemon plants (Table 4). Interestingly, numerous genes encoding glutathione transferases (GSTs) belonging to different GST classes have been induced by the fungal infection. This gene family can positively contribute to antimicrobial resistance in host plants by mostly unknown mechanisms, although a recognized GST function is their participation in the elimination of ROSs and lipid hydroperoxides that accumulate in infected tissues [42,43].

##### Light Reactions

As shown in Table 4 and Figure S6, the light reactions of the photosynthetic pathway were strongly affected by *P. tracheiphilus* inoculation as most of the components of both light harvesting and photosynthetic electron flows (cyclic and non-cyclic) as well as subunits of the CF0F1-ATP synthase were down regulated in inoculated plants (Figure S6). In detail, the PSAE-2 photosystem I subunit E-2 and the PSBE photosystem II reaction center protein as well as thylakoid-associated phosphatase 38 (Table 4) were down regulated in seedlings the diseased plant. This last gene is involved in light-harvesting complex of photosystem II (LHCII) dephosphorylation, facilitating its relocation to photosystem I. The expression of NDH-dependent cyclic electron flow 1 complex, that is involved in the cyclic electron transport by accepting electrons from ferredoxin (Fd), was sharply repressed. Moreover, the expression of the CF1-ATP synthase subunit was downregulated suggesting that the photophosphorylation of ADP leading to the ATP synthesis is strongly impaired because of fungal infection. Considering that photosynthesis is the main metabolic pathway devoted to energy supply in the green part of the plants, these findings clearly indicate that inoculated plants were suffering of energy shortage.

##### Iron Homeostasis

As shown in Table 4, genes involved in iron uptake and reduction were differently regulated in the *Pt* vs. CK comparison. In particular, ferric reduction oxidase 6 (FRO6), FRO7 and FRO8 were repressed by the infection. These genes are proposed to be involved in iron transport across the membrane in green part of the plant, FRO6 being localized in the plasma membrane, FRO7 in the chloroplasts and FRO8 in mitochondria [44]. These results clearly indicate that the iron homeostasis is sharply impaired in the organelles of inoculated plants and in chloroplasts where it plays a crucial role in the heme biosynthesis and photosynthesis. Ferric reduction oxidase 2 (FRO 2) and 4 (FRO4) which normally are expressed in plant roots were upregulated by fungal infection, as well as the gene encoding 2-oxoglutarate (2OG) and Fe(II)-dependent oxygenase which are involved in sideretin biosynthesis, a metabolite exuded by roots in response to iron deficiency to facilitate iron uptake. The stress induced expression of genes, both FROs and 2OG, normally involved in iron uptake in roots might be explained as an ultimate attempt to cope with the shoot iron deficiency caused by the down regulation of leaf-specific FRO genes. Regarding other nutrients such as nitrogen and phosphate, the results show that gene involved in nitrate uptake was down-regulated, whereas glutamine synthase and aspartate aminotransferase involved in nitrogen fixation and in amino acid and Krebs cycle metabolisms were up-regulated. The high-affinity transporter for external inorganic phosphate functioning as H^+^: Phosphate symporter was also up-regulated (Table 4).

##### Carbohydrate Metabolism

The analysis of carbohydrate metabolism highlighted that several genes involved in sugar metabolism were clearly induced in response to fungal infection (Table 4). Specifically, sucrose-phosphate synthase 4, which plays a role in photosynthetic sucrose synthesis by catalyzing the rate-limiting step of sucrose biosynthesis from UDP-glucose and fructose- 6-phosphate, was down-regulated. On the contrary, transcripts encoding sucrose synthase, a cleaving enzyme that provides UDP-glucose and fructose for various metabolic pathways, were among the up-regulated genes. Table 4 also reports that transcripts encoding the acid beta-fructofuranosidase and alkaline/neutral invertase, respectively involved in the continued mobilization of sucrose to sink organs and in the cleavage of sucrose into glucose and fructose, were up-regulated. Overall, these data suggest that both sucrose synthesis and therefore the export of photo assimilates out of the leaf were impaired, whereas cleavage seems to be the favorite route undertaken by this metabolite. However, the fructose-bisphosphate aldolase 1 and hexokinase 1 were down-regulated in diseased plants indicating that glycolysis might be repressed in the inoculated plants (Table 4).

##### Cell Wall Modification and Degradation

During pathogen infections, the cell wall undergoes dramatic structural and chemical changes of cell wall constituents. Necrotrophic pathogens are sensed by a plasma membrane receptor, leading to activation of defense signaling cascades and eventual mounting of inducible defense responses [45]. In our study, several DEGs encoding pectin lyase-like superfamily protein and pectin acetylesterases were identified (Table 4). However, these transcripts were both up- and down-regulated, making it difficult to extrapolate unequivocal conclusions. Certainly, as expected, cell walls of inoculated plants underwent remodeling processes likely involved in the response to pathogen.

## 3. Discussion

Environmental stresses severely affect plant and crop growth and reproduction. Therefore, determining the critical molecular mechanisms and cellular processes in response to stresses will provide knowledge for identifying genes that might be target of modification, by *knocking out* or by *knocking down* procedures, especially in susceptible host–pathogen interactions. RNA sequencing (RNA-Seq) using next-generation sequencing (NGS) provides opportunity to isolate genes of interest, develop of functional markers, quantify of gene expression and carry out comparative genomic studies. It has been successfully applied to unravel the transcriptome profile of several *Citrus* varieties in response to *Phytophtora parasitica* infection [26] and to *Candidatus* Liberibacter [24,25] providing new insight into host responses to both pathogens. In this work, we described the results of RNA sequencing and de novo transcript assembly in rough lemon (*C. jambhiri*) leaves subjected to artificial inoculation by *P. tracheiphilus*, the causal agent of “mal secco” disease used as model of a compatible host–pathogen interaction. At harvest time (15 days after inoculation), infected plants showed the typical disease symptoms, and the pathogen was detected by molecular analysis. Globally, a deep reprogramming of the leaf transcriptome emerged as 4986 (2865 up-regulated and 2121 down regulated) DEGs have been identified confirming that the attempt of an active defense against microbial pathogens involved the induction of elaborate defense signaling pathways. In plants, some of these defense strategies can provide protection at the site of infection, whereas others provide systemic resistance throughout the plant including in non-infected tissue. Local resistance includes basal immunity, or PAMP/MAMP (pathogen/microbe associated molecular patterns)—triggered immunity (PTI) which is induced when pattern recognition receptors (PRRs) from the plant recognize pathogen-derived elicitors. To establish a successful infection, plant pathogens can suppress PTI by injecting effectors into the host cells [46]. To counter this virulence strategy, plants have evolved the so-called resistance (R) proteins, which can either directly detect the effectors or indirectly detect their activity. In plants where the activity of effectors is detected by the R proteins, effector-triggered immunity (ETI) is activated rendering the pathogen avirulent [47] ETI in plants is often associated with rapid, localized programmed cell death (PCD) at the infection site, a visible phenotype known as the hypersensitive response HR, to prevent the spread of the pathogen. HR is generally associated with race-specific resistance to biotrophic pathogens and it is less effective against necrotrophics which require dead host tissue to complete their life cycle [47]. Necrotrophic pathogens such as *P. tracheiphilus* are well able to block HR by initiating systemic signals for defense activation in distal parts of plant that ultimately results in the activation of systemic acquired resistance (SAR) [47]. Induction of SAR involves the generation of mobile signals at the site of primary infection, which translocate to distal tissue and prepare the plant against future infections. Several chemical inducers of SAR have been identified and some of these have been shown to translocate systemically. The SAR associated chemicals include salicylic acid (SA), free radicals, and reactive oxygen species (ROS), among others [48]. Upon SA accumulation, NPR1 monomers are transported into the nucleus. Here, NPR1 interacts with TGA proteins, which belong to the basic leucine zipper (bZIP) protein family of transcription factors and binds TGACG motifs to activate defense-related transcription [48]. The analysis of the transcriptomic data reported in this work unequivocally indicated that the entire gene set encoding the components of SAR from salicylic acid biosynthesis on was strongly up-regulated. In addition, *P. tracheiphilus* is able to overcome the basal immunity of rough lemon plants (PTI) as the essential regulator of plant defense (RPM1 interacting protein 4) was down-regulated, and the expression of BIR2, which is negative regulator of basal level of immunity was up-regulated in the diseased plants. In the inoculated plants, the observed repression of auxin signaling by the SA pathway might also contribute to increase rough lemon susceptibility to *P. tracheiphilus* as reported in *Arabidopsis* infected by the necrotrophic fungi *Plectosphaerella cucumerina* and *Botrytis cinerea* [49].

Chitin, found in the cell walls of true fungi, is a well-established elicitor of plant defense responses and it appears to play a significant role in plant defense to fungal pathogens [50]. The fact that chitin elicits de novo gene expression suggests the involvement of transcription factors (TFs) with WRKY TF family strongly represented [51,52,53]. To regulate gene expression, WRKY proteins bind specifically to a DNA sequence motif (T)(T)TGAC(C/T) known as the W box [54,55,56,57] which occurs in the promoters of genes under the control of WRKY proteins. A number of defense-related genes, including PR genes, contain a W box in their promoter regions [51,54,55]. The promoters of pathogen and/or salicylic acid (SA) regulated *Arabidopsis* WRKY genes [58] are substantially enriched in W boxes, suggesting that defense-regulated expression of WRKY genes involves transcriptional activation and repression through self-regulatory mechanisms mediated by transcription factors of the WRKY gene superfamily [32]. For example, expression of the *Arabidopsis NPR1* is known to be controlled by WRKY factors [57]. In this study, transcription factors interacting specifically with the W box motif such as *WRKY14*, *WRKY23*, *WRKY49*, *WRKY72*, *WRKY75*, and *WRKY71* were up-regulated in infected plants, indicating a strong activation of the defensive mechanism. The up-regulation of both *WRKY4*, that is reported to have a positive role in plant resistance to necrotrophic pathogens [34] and *WRKY51*, acting as positive regulator of salicylic acid (SA)-mediated signaling [33] confirms that the rough lemon plants tried to resist the *P. tracheiphilus* infection by the activation of salicylic acid-mediated signaling pathway, in accordance with the whole results of this study. Furthermore, the strong induction of *WRKY40*, *WRKY18,* and *WRKY70* transcription factors accounts for a defense response specifically addressed towards fungi as they specifically respond to chitin [31]. *P. tracheiphilus* infection induced the expression of oxidative burst peroxidases (RBOHs) in rough lemon (Table 3). The apoplastic oxidative burst could directly kill pathogens by generating ROS with antimicrobial activity; otherwise, a second, stronger phase can occur, which is associated with the hypersensitive response [39]. However, the role of RBOHs is controversial as a relatively limited role for NADPH oxidases in the HR has been observed in tobacco (*Nicotiana tabacum*), where RBOHD-mediated hydrogen peroxide production does not seem to be essential for the development of the HR or systemic acquired resistance (SAR) [59,60]. More recently, these genes have been studied in detail in *A. thaliana* and are reported as the major component of PTI [39]. Considering that rough lemon plant is susceptible to *P. tracheipilus*, their effectiveness in overcoming the pathogen is not sufficient to block it, and probably they have a major role in transducing the signal of the “presence” of the pathogen by increasing ROS concentration.

Although important in biotic stress signal transduction, reactive oxygen species (ROS) such as hydrogen peroxide (H_2_O_2_), superoxide (O^2−^), and singlet oxygen (^1^O_2_) are highly reactive and could cause oxidative damage to DNA, proteins and other molecules of the cell. There are different cellular mechanisms in place to deactivate the excess of these damaging ROS molecules. These include enzymatic reactions through catalase, superoxide dismutase, glutathione peroxidase and ascorbate peroxidase but also small antioxidants such as ascorbic acid and glutathione [61]. This study revealed that a subset of these ROS-scavenging genes was induced in the infected plants (Table 4). Interestingly, a wide number of glutathione transferases belonging to phi and tau classes were also up-regulated by the infection in accordance with early studies on the role of GSTs in plant biotic stress [43]. Notably, the expression of multiple GSTs was massively activated by salicylic acid and some GST enzymes were demonstrated to be receptor proteins of salicylic acid [43]. Functional studies revealed that overexpression or silencing of specific GSTs can markedly modify disease symptoms and pathogen multiplication rates [62].

As reported in the case of other necrotrophic fungi such as *B. cinerea* [63], the main metabolic effect upon inoculated plants was the down-regulation of either light harvesting components or photosynthetic electron flows or CF1F0-ATPase. This might have led to an apparent, persistent “dark” or “shade” condition: Plants are in regular light/dark alternation but they cannot use light to provide energy. The sucrose mobilization suggested by the regulation of the two main genes involved in sucrose biosynthesis and cleavage is in accordance with this energy requirement. In the dark, plant mitochondria generate the required ATP molecules for basic cellular function [64]. However, two main genes involved in glucose catabolism were down-regulated (Table 4) indicating that sugars seem not be routed towards glycolysis and Krebs cycle. On the contrary, as fungal genes involved either in sugar fermentation or in mitochondrial synthesis of ATP were strongly expressed in rough lemon leaves, the plant sugar resources might be hijacked towards the fungus to feed it. This mode of nutrition is the rule for biotrophic pathogens, but also necrotrophics might exhibit a similar behavior [65]. In this study, we also show that FRO7 (chloroplast Fe(III) chelate reductase), involved in chloroplast iron homeostasis and required for survival under iron-limiting conditions, was down regulated. It has been shown that chloroplasts isolated from *fro7* loss-of-function mutant plants have significantly reduced Fe(III) chelate reductase activity, reduced iron content, and altered photosynthetic complexes, providing genetic proof that chloroplasts do rely in part on a reductive strategy for iron acquisition [44]. Consequently, the lack of a regular input of reducing power from water photolysis induced by light might be in turn responsible for the iron deficiency observed in the apical part of the leaves of diseased rough lemon.

## 4. Materials and Methods

### 4.1. Plant Material and Inoculum

Seeds of rough lemon (*C. jambhiri*) were sowed on sterile peat in May 2019. After 6 months of growing in a chamber at 25 °C and 90% humidity, the plants were inoculated with the pathogen *Plenodomus tracheiphilus* PT10 strain (kindly provided by Professor Vittoria Catara, University of Catania). Rough lemon was chosen as plant material for the following reasons: (I) It was previously reported as very susceptible to the disease [6]; (II) it has a high degree of polyembryony, higher than true lemons or other citron hybrids [66], allowing the production of true-to-type seedlings; and (III) it is very vigorous, with seedlings growing faster than those of other citrus species. Moreover, our preliminary inoculation tests indicated that symptoms after artificial inoculations were easier to detect in rough lemon than in *C. limon* seedlings.

The inoculum was prepared according to a slight modification of the method described in [67]. Briefly, three pieces of young fungus grown at 21 °C ± 2 in Petri dishes containing potato dextrose agar medium (PDA) were placed in 7 different flasks containing 100 mL of carrot broth and incubated for 5 days in a heidolph unimax 2010 shaker at 22 °C. Successively, the growth medium was filtered and centrifugated at 8000 rpm × 20 min. The pellet was recovered and the phialoconidia were counted with a counting chamber to adjust the inoculum concentration at 10^6^ mL^−1^. The inoculation was performed by depositing 10 µL on wounds obtained by cutting the midvein of three leaves for each plant with a sharp sterile blade. Overall, five plants were inoculated with the pathogen and five plants were inoculated with water as control. Both inoculated and control samples were collected 15 days after inoculation, considering that inoculated plants showed evident symptoms of the disease. Leaves were immediately frozen with liquid nitrogen and stored at −80 °C until both DNA and RNA extractions were performed.

### 4.2. DNA and RNA Extraction

DNA extraction was performed according to [68]. Briefly, samples were powdered using liquid nitrogen in mortar and pestle. Two hundred milligrams of grinded leaves were mixed approximately with 500 μL of CTAB buffer (2% CTAB, 20 mM EDTA, 1.44 mM NaCl, 100 mM Tris HCl, pH 8.0) and 0.2% β-mercaptoethanol. Samples were vortexed and incubated at 65 °C for 30 min, then the CTAB-plant extract mixture was transferred into a microfuge tube. After adding 300 µL of chloroform-isoamyl alcohol (24/1), the tubes were mixed by inversion and centrifuged at 14,000 rpm for 10 min. The supernatant was recovered into a clean microfuge tube and 7.5 M ammonium acetate (50 μL) followed by 1000 μL of ice cold 100% of ethanol were added to each tube. The tubes were mixed by inversion and then centrifuged at 10,000 rpm for 10 min. The pellet was rinsed twice with 1000 µL of ice cold 70% ethanol, resuspended in 50 µL of sterile distilled water and stored at 4 °C until analysis. The DNA concentration and purity were checked by a Nanodrop 2000 spectrophotometer (Thermo Scientific™, Waltham, MA, USA).

The RNA was extracted using the RNeasy^®^ Plant Mini Kit (Qiagen, Venlo, The Netherlands) according to the manufacturer’s instructions. RNA degradation and contamination were monitored on 1% agarose gels. RNA purity and concentration were checked using the NanoDrop spectrophotometer (ThermoFisher Scientific, Waltham, MA, USA). Before sequencing, sample RNA integrity (RIN) was assessed using the Agilent Bioanalyzer 2100 system (Agilent Technologies, Santa Clara, CA, USA).

### 4.3. Real-Time Confirmation of Infected Plants

Taqman Real-time PCR was performed to reveal the presence of the pathogen within the inoculated plants using an ABI 7500 Real-Time PCR System (Applied Biosystems™, Foster City, CA, USA). The analysis was performed according to the method described in [28], using DNA extracted from both inoculated and control leaves as template. Forward primer GR70 (5′-GATCCGTACGCCTTGGGGAC-3′) and reverse primer, GL1 (5′-AGAAGC GTTTGGAGGAGAGAATG-3′), dual-labeled fluorogenic probe, PP1, (5′-FAM-CACGCAATCTTGGCGACTGTCGTT-TAMRA-3′) were used with the aim to amplify a 84-bp segment of the pathogen DNA. Each reaction contained 200 nΜ forward primer, 200 nM reverse primer, 100 nM fluorogenic probe, and 4 µL of genomic DNA in a final volume of 15-µL. Negative control contained the same mixture, with sterile water replacing the DNA template. The assay was performed on three biological replicates, each one repeated twice. The thermal cycling conditions for *P. tracheiphilus* DNA template amplification were 50 °C for 2 min (1 cycle), 95 °C for 30 s (1cycle), 95 °C for 10 s, 62 °C for 30 s (40 cycles). Standard curve for fungal DNA quantification was constructed using *P. tracheiphilus* DNA (100 µg mL^−1^) extracted from the Pt10 strain and serially diluted in sterile distilled water as described in [28].

### 4.4. Library Preparation and Sequencing

After the QC procedures, sequencing libraries were generated using NEBNext^®^ Ultra™ RNA Library Prep Kit for Illumina^®^ (New England Biolabs, Ipswich, MA, USA) following manufacturer’s recommendations and as reported in [69]. Briefly, mRNA was enriched using poly-T oligo-attached magnetic beads. Fragmentation was carried out using divalent cations under elevated temperature in NEBNext First Strand Synthesis Reaction Buffer (5X), followed by cDNA synthesis using random hexamers and M-MuLV Reverse Transcriptase (RNase H-). After first-strand synthesis, a custom second-strand synthesis buffer (Illumina) was added containing dNTPs, RNase H and *Escherichia coli* polymerase I to generate the second strand by nick-translation. After adenylation of 3′ ends of DNA fragments, NEBNext Adaptor with hairpin loop structure were ligated to prepare for hybridization. To select cDNA fragments preferentially 150~200 bp in length, the library fragments were purified with AMPure XP system (Beckman Coulter, Beverly, MA, USA). Then, 3 μL USER Enzyme by NEB was used with size-selected, adaptor-ligated cDNA at 37 °C for 15 min followed by 5 min at 95 °C before PCR. Successively, PCR was performed with Phusion High-Fidelity DNA polymerase, Universal PCR primers and Index (X) Primer. Library concentration was first quantified using a Qubit 2.0 fluorometer (Life Technologies, Carlsbad, CA, USA), and then diluted to 1 ng/µL before checking insert size on an Agilent Bioanalyzer 2100 system (Agilent Technologies, Santa Clara, CA, USA). Cluster generation and sequencing were performed by Novogene Bioinformatics Technology Co., Ltd. (Beijing, China). After cluster generation, the libraries were sequenced on Illumina HiSeq2000 platform to generate pair-end reads. Raw data (raw reads) of fastq format were firstly processed through in-house perl scripts. In this step, clean data were obtained by removing reads containing adapters, reads containing poly-N and low-quality reads. Sequences putatively belonging to pathogen in inoculated rough lemon samples were removed by filtering out the reads mapped to the fungus genome (https://mycocosm.jgi.doe.gov/Photr1/Photr1.info.html, accessed on 18 November 2020). Then Q20, Q30, GC-content and sequence duplication level of the clean data were calculated. All the downstream analyses were based on clean data with high quality.

### 4.5. De novo Transcriptome Assembling and Gene Functional Annotation

De novo transcriptome assembly was accomplished using Trinity (r20140413p1 version) with min_kmer_cov:2 parameters (k = 25). Then Hierarchical Clustering was performed by Corset (v1.05 version, https://github.com/Oshlack/Corset/wiki) to remove redundancy (parameter -m 10) and the longest transcripts of each cluster were selected as Unigenes. The flow chart of the rough lemon de novo transcriptome assembly is stackable to that reported in [69]. The *Citrus jambhiri* transcriptome was uploaded to NCBI (https://www.ncbi.nlm.nih.gov/geo/, accessed on 29 December 2020) accession number GSE164096.

Gene function was annotated based on the following databases: National Center for Biotechnology Information (NCBI) non-redundant protein sequences (Nr), NCBI non-redundant nucleotide sequences (Nt), Protein family (Pfam), Clusters of Orthologous Groups of proteins (KOG/COG), Swiss-Prot, Kyoto Encyclopedia of Genes and Genomes (KEGG), Ortholog database (KO) and Gene Ontology (GO). A pathway analysis was conducted using MapMan3.6.0RC1 (https://mapman.gabipd.org/, accessed on 19 October 2020). All the unigenes were annotated and mapped using Mercator4 V2.0, an on-line tool of MapMan (https://www.plabipd.de/portal/mercator4, accessed on 5 November 2020) which accurately assigns hierarchal ontology providing visual representation of genes in different plant processes. The significant DEGs (padj < 0.05), with the corresponding log_2_FoldChange values, were used as dataset to align with the Mercator map.

### 4.6. Quantification of Gene Expression and Differential Expression Analysis

Gene expression levels were estimated by RSEM (v1.2.26 version, http://deweylab.github.io/RSEM/) with bowtie2 mismatch 0 parameters to map the Corset filtered transcriptome. For each sample, clean data were mapped back onto the assembled transcriptome and readcount for each gene was then obtained. Differential expression analysis between control (CK) and infected (*Pt*) samples was performed using the DESeq R package (1.12.0 version, padj < 0.05, https://bioconductor.org/packages/release/bioc/html/DESeq.html). The resulting *p*-values were adjusted using the Benjamini and Hochberg’s approach for controlling the false discovery rate [70]. Genes with an adjusted *p*-value < 0.05 found by DESeq were assigned as differentially expressed. A log_2_FoldChange threshold of 0.58 (1.5 fold change) was adopted. The GO enrichment analysis of the differentially expressed genes (DEGs) was implemented by the GOseq R packages (1.10.0, 2.10.0 version, corrected *p* value < 0.05 based) Wallenius non-central hyper-geometric distribution. Furthermore, all of the unigenes were submitted to the KEGG pathway database for the systematic analysis of gene functions. KOBAS software (v2.0.12 version, corrected *p*-Value < 0.05, kobas.cbi.pku.edu.cn) was used to test the statistical enrichment of differential expression genes in KEGG pathways.

### 4.7. Real-Time Validation of Selected DEG Candidates Using qRT-PCR

Total RNA (2.5 μg) extracted from sample leaves as described above, was reversed transcribed using the SuperScript™ Vilo™ cDNA synthesis kit by ThermoFisher Scientific (Warrington WA1 4SR, UK), according to the manufacturer’s instructions. Real-time qRT-PCR was performed for a total of 10 DEGs with PowerUp SYBR Green Master mix by ThermoFisher Scientific and carried out in the Bio-Rad iQ5 Thermal Cycler detection system. All the genes were normalized with *Citrus clementina* actin (LOC18039075). All reactions were performed in triplicate and fold change measurements calculated with the 2^−ΔΔCT^ method. The selected DEGs and their corresponding primer sequences are provided in Table S2.

## 5. Conclusions

The global transcriptome analysis of *Pt* vs. control plants led to the identification of genes and metabolic pathways involved in rough lemon response to *P. tracheiphilus*. As far as we know, this is the first manuscript that describes at molecular level the “mal secco” disease induced by *P. tracheiphilus* in citrus and makes *C. jambhiri* genetic resources available for the scientific community interested in citrus breeding.

The results highlight most of the events occurring during this compatible host–pathogen interaction, which now it is known relies on the activated SA signal cascade that, in turn, induces systemic acquired resistance (SAR). As the main scope of the work was the identification of putative target genes for genome editing experiments, a wide range of genes belonging to structural and transcription factors have been identified and they could be taken in consideration for targeted mutagenesis, RPM1 and BIR2 being only two of them. This strategy fits the increased demand for economical and environmentally friendly approaches to cope with plant diseases, while avoiding the use of agrochemicals.

## Figures and Tables

**Table 1:** Summary statistics of the RNA quality and sequencing results.

Parameter	Value
Average RIN	8.2
Clean reads	228 million
N° of transcripts	115,100
N° of Unigenes	77,631
Average of read mapped rate	83.40%
Transcripts N50 (bp)	2372
Unigenes N50 (bp)	2060
Q30 (%)	92.82
GC content (%)	44.22

**Table 2:** List of “Plant hormone” related DEGs identified in *Pt* vs. CK comparison.

Cluster	Symbol	Annotation	TAIR Code	Log_2_Fold Change	Identity Score	e-Value
*Auxin*						
5112,0	YUC6	Flavin-binding monooxygenase family protein	AT5G25620	−4.22	69%	4 × 10^−^^69^
15782,1	AUX1	Transmembrane amino acid transporter family protein	AT2G38120	−5.18	74%	0.0
14701,68946	TIR1	F-box/RNI-like superfamily protein	AT3G62980	−2.62	66%	2 × 10^−^^26^
10078,0	IAA18	Indole-3-acetic acid inducible 18	AT1G51950	−3.18	79%	7 × 10^−^^9^
16281,1	IAA4	AUX/IAA transcriptional regulator family protein	AT5G43700	−2.91	75%	3 × 10^−^^63^
16862,0	IAA32	Indole-3-acetic acid inducible 32	AT2G01200	−3.39	70%	3 × 10^−^^20^
14701,19495	IAA7	Indole-3-acetic acid 7	AT3G23050	−5.42	83%	5 × 10^−^^71^
16281,0	IAA3	AUX/IAA transcriptional regulator family protein	AT1G04240	−3.61	81%	0.001
14701,30415	ARF3	Auxin-responsive factor AUX/IAA-related	AT2G33860	+4.30	73%	2 × 10^−^^168^
14701,26809	GH3.3	Auxin-responsive GH3 family protein	AT2G23170	+3.42	72%	5 × 10^-123^
17976,0		SAUR-like auxin-responsive protein family	AT2G36210	+3.41	82%	3 × 10^−^^4^
*Ethylene*						
20624,0	ACS2	1-amino-cyclopropane-1-carboxylate synthase 2	AT1G01480	+7.94	68%	2 × 10^−^^131^
14701,32226	ACO1	ACC oxidase 1	AT2G19590	+6.30	72%	9 × 10^−^^133^
17499,2	ACS6	1-aminocyclopropane-1-carboxylic acid (acc) synthase 6	AT4G11280	+5.09	69%	4 × 10^−^^139^
14701,57239	ETR2	Signal transduction histidine kinase, hybrid-type, ethylene sensor	AT3G23150	+3.46	66%	2 × 10^−^^170^
14701,58523	EBF1	EIN3-binding F box protein 1	AT2G25490	+8.86	66%	2 × 10^−^^87^
14701,46599	EIN3	Ethylene insensitive 3 family protein	AT3G20770	+7.72	76%	0.0
6645,0	ERF1	Ethylene response factor 1	AT3G23240	+3.32	72%	6 × 10^−^^58^
14701,24495	ERF2	Ethylene responsive element binding factor 2	AT5G47220	+2.49	76%	3 × 10^−^^46^
14701,21798	ERF13	Ethylene-responsive element binding factor 13	AT2G44840	+2.53	78%	1 × 10^−^^37^
14701,35256	ERF4	Ethylene responsive element binding factor 4	AT3G15210	+2.42	78%	2 × 10^−^^36^
14701,7830	ERF110	Ethylene response factor 110	AT5G50080	+4.23	77%	6 × 10^−^^28^
*Salicylic acid*						
14701,45136	PAL1	Phenylalanine ammonia lyase 1	AT2G37040	+3.82	74%	0.0
14701,67897	4CL	4-coumarate–CoA ligase-like 5	AT1G51680	+3.89	72%	1 × 10^−^^30^

**Table 3:** List of defense and pathogenesis related DEGs identified in *Pt* vs. CK comparison.

Cluster	Symbol	Annotation	TAIR Code	Log_2_Fold Change	Identity Score	e-Value
*Response to pathogen*						
14701,26919	CPK33	calcium-dependent protein kinase 33	AT1G50700	+3.13	67%	4 × 10^−^^22^
14701,66288	CRCK3	calmodulin-binding receptor-like cytoplasmic kinase 3	AT2G11520	+2.33	71%	5 × 10^−^^59^
17682,2	CML11	calmodulin-like 11	AT3G22930	+3.33	76%	2 × 10^−^^77^
14701,33952	MPK3	mitogen-activated protein kinase 3	AT3G45640	+5.42	76%	2 × 10^−^^28^
14701,16139	MAPKKK15	mitogen-activated protein kinase kinase kinase 15	AT5G55090	+2.49	67%	1 × 10^−^^58^
20990,0	MAPKKK17	mitogen-activated protein kinase kinase kinase 17	AT2G32510	+3.46	64%	1 × 10^−^^33^
14701,65619	CERK1	chitin elicitor receptor kinase 1	AT3G21630	+5.56	75%	3 × 10^−^^18^
8490,0	PR1	pathogenesis-related gene 1	AT2G14610	+4.09	68%	8 × 10^−^^31^
14701,26429	PRB1	basic pathogenesis-related protein 1	AT2G14580	+8.96	68%	2 × 10^−^^32^
16905,0	NPR1	regulation of innate immune response		+5.40		
13144,0	NPR1	*Citrus sinensis* protein NIM1-INTERACTING 3 (LOC107177379)		+4.21	100%	0.0
18290,0	CF-9	*Citrus clementina* receptor-like protein 9DC3 (LOC18042467)		+5.75	97%	3 × 10^−^^121^
6996,2	BIR2	Inactive LRR receptor-like serine/threonine-protein kinase	AT3G47570	+5.22	98%	0.0
14701,81960	CES101	lectin protein kinase family protein	AT3G16030	+5.49	77%	7 × 10^−^^11^
14701,15619	IOS1	Leucine-rich repeat transmembrane protein kinase protein	AT2G19230	+5.15	68%	2 × 10^−^^16^
14701,38537	EIX2	*Citrus sinensis* receptor-like protein EIX2 (LOC102609951)		+4.79	80%	0.0
14701,79574	LECRK3	*Citrus clementina* G-type lectin S-receptor-like serine/threonine-protein kinase (LOC18049964)		+4.20	99%	0.0
14701,84653	RGS1	G-protein coupled receptors; GTPase activators	AT3G26090	+4.76	70%	4 × 10^−^^81^
14701,13865	TGA2	transcription factor TGA2.3 isoform X1	AT5G06950	+3.44	70%	5 × 10^−^^102^
14701,40930	BAD1	Ankyrin repeat family protein BAD1	AT1G14500	+3.18	75%	2 × 10^−^^7^
19125,1	RIN4	RPM1 interacting protein 4	AT3G25070	−4.22	69%	2 × 10^−^^14^
14701,27598	RBOHF	respiratory burst oxidase protein F	AT1G64060	+2.41	75%	0.0
14701,78394	RBOHB	respiratory burst oxidase homolog B	AT1G09090	+6.89	72%	4 × 10^−^^154^
14701,77930	RBOHC	NADPH/respiratory burst oxidase protein D	AT5G51060	+3.51	72%	1 × 10^−^^8^
14701,55000	RBOHD	respiratory burst oxidase homologue D	AT5G47910	+2.74	72%	0.0
*WRKY transcription factors*						
16089,0	WRKY35	WRKY DNA-binding protein 35	AT2G34830	+2.39	83%	1 × 10^−^^92^
15844,0	WRKY49	WRKY DNA-binding protein 49	AT5G43290	+2.89	77%	1 × 10^−^^8^
14701,6540	WRKY23	WRKY DNA-binding protein 23	AT2G47260	2.78	76%	5 × 10^−^^80^
14701,12356	WRKY4	WRKY DNA-binding protein 4	AT1G13960	+2.35	77%	5 × 10^−^^24^
21223,0	WRKY72	WRKY DNA-binding protein 72	AT5G15130	+5.75	82%	7 × 10^−^^87^
14701,60912	WRKY50	WRKY DNA-binding protein 50	AT5G26170	+5.31	78%	3 × 10^−^^36^
14701,18458	WRKY40	WRKY DNA-binding protein 40	AT1G80840	+5.28	74%	5 × 10^−^^27^
16962,0	WRKY75	WRKY DNA-binding protein 75	AT5G13080	+5.07	76%	4 × 10^−^^53^
14701,3630	WRKY71	WRKY DNA-binding protein 71	AT1G29860	+4.71	84%	2 × 10^−^^24^
14701,66972	WRKY18	WRKY DNA-binding protein 18	AT4G31800	+4.34	77%	2 × 10^−^^17^
14701,51257	WRKY70	WRKY DNA-binding protein 70	AT3G56400	+4.03	72%	1 × 10^−^^18^
14701,2889	WRKY44	WRKY family transcription factor family protein	AT2G37260	−2.43	80%	2 × 10^−^^43^

**Table 4:** List of DEGs identified in *Pt* vs. CK comparison.

Cluster	Symbol	Annotation	TAIR Code	Log_2_Fold Change	Identity Score	e-Value
*Reactive oxygen—Oxidoreductase activity*						
14701,18284	CSD1	Copper/zinc superoxide dismutase 1	AT1G08830	+9.31	69%	2 × 10^−^^4^
14701,15083	APX2	Ascorbate peroxidase 2	AT3G09640	+5.11	78%	2 × 10^−^^166^
14701,14158	PMP22	Peroxisomal membrane 22 kDa	AT4G04470	−4.28	80%	2 × 10^−^^30^
14701,8276	AOS	Allene oxide synthase	AT5G42650	+9.59	67%	6 × 10^−^^26^
14701,29676	ALDH3H1	Aldehyde dehydrogenase 3H1	AT1G44170	+3.70	68%	3 × 10^−^^6^
*Reactive oxygen—Glutathione metabolism*						
14701,7488	GSTU19	Glutathione S-transferase TAU 19	AT1G78380	+7.58	70%	1 × 10^−^^56^
14701,35413	GSTU10	Glutathione S-transferase TAU 10	AT1G74590	+5.23	73%	1 × 10^−^^17^
14701,48103	GSTF9	Glutathione S-transferase PHI 9	AT2G30860	+5.22	72%	1 × 10^−^^53^
14701,17358	GSTU7	Glutathione S-transferase TAU 7	AT2G29420	+4.54	71%	1 × 10^−^^33^
14701,48102	GSTF9	Glutathione S-transferase PHI 9	AT2G30860	+4.32	69%	3 × 10^−^^5^
*Light reaction*						
14701,61813	PSAE-2	Photosystem I subunit E-2	AT2G20260	−2.58	77%	1 × 10^−^^6^
14701,4480	PSBE	Photosystem II reaction center protein E	ATCG00580	−2.31	95%	9 × 10^−^^110^
14701,34255	ATPD	ATP synthase delta-subunit gene	AT4G09650	−3.10	72%	2 × 10^−^^59^
14701,26690	PSBS	Chlorophyll A-B binding family protein	AT1G44575	−3.50	76%	2 × 10^−^^24^
14701,72032	TAP38	Thylakoid-associated phosphatase 38	AT4G27800	−3.42	68%	5 × 10^−^^42^
14701,83115	FKBP16	FK506-binding protein 16-2	AT4G39710	−5.55	75%	2 × 10^−^^90^
14701,66882	NDF4	NDH-dependent cyclic electron flow 1 complex	AT3G16250	−7.12	74%	1 × 10^−^^61^
14701,65295	NDHB.2	NADH-Ubiquinone/plastoquinone (complex I) protein (chloroplastic)	ATCG01250	−2.44	98%	0.0
14701,64497	PPDK	Pyruvate orthophosphate dikinase (chloroplastic)	AT4G15530	−2.79	77%	0.0
*Nutrient homeostasis*						
14701,19268		2-oxoglutarate (2OG) and Fe(II)-dependent oxygenase superfamily protein	AT1G55290	+7.77	68%	2 × 10^−^^86^
14701,28407	FRO4	Ferric reduction oxidase 4	AT5G23980	+7.14	66%	2 × 10^−^^120^
19914,4	FRO2	Ferric reduction oxidase 2	AT1G01580	+3.94	69%	1 × 10^−^^36^
14701,21908	FRO7	Ferric reduction oxidase 7 (chloroplastic)	AT5G49740	−3.93	73%	1 × 10^−^^81^
14701,21905	FRO6	Ferric reduction oxidase 6	AT5G49730	−4.33	75%	3 × 10^−^^105^
14701,82040	FRO8	Ferric reduction oxidase 8 (mithocondrial)	AT5G50160	−5.65	69%	9 × 10^−^^106^
4412,0	IREG2	Iron regulated 2	AT5G03570	−5.78	79%	6 × 10^−^^40^
14701,68697	ASP3	Aspartate aminotransferase 3 (chloroplastic)	AT5G11520	+2.74	80%	0.0
14701,45698	GLN1;1	Glutamine synthase clone R1 (cytosolic isozyme 1)	AT5G37600	+2.66	77%	0.0
20088,0	NRT2:1	Nitrate transporter 2:1	AT1G08090	−2.41	73%	2 × 10^−^^111^
14701,24935	PHT1;4	Phosphate transporter 1;4	AT2G38940	+2.78	73%	0.0
*Carbohydrate metabolism*						
14701,30461	SUS2	Sucrose synthase 2	AT5G49190	+5.52	79%	7 × 10^−^^63^
14701,11795	SUS6	Sucrose synthase 6	AT1G73370	+2.33	71%	0.0
14701,60145	SPS4F	Sucrose-phosphate synthase 4	AT4G10120	−2.33	82%	4 × 10^−^^29^
14701,28539	BETAFRUCT4	Acid beta-fructofuranosidase	AT1G12240	+8.66	70%	0.0
14701,71035	INV-E	Alkaline/neutral invertase (chloroplastic)	AT5G22510	+2.36	72%	6 × 10^−^^162^
14701,25319	FBA1	Fructose-bisphosphate aldolase 1	AT2G21330	−3.27	74%	8 × 10^−^^10^
14701,77303	HXK1	Hexokinase 1	AT4G29130	−3.81	73%	2 × 10^−^^97^
*Cell wall modification and degradation*						
11195,0	QRT3	Pectin lyase-like superfamily protein	AT4G20050	+6.07	67%	2 × 10^−^^38^
14701,45234		Pectinacetylesterase family protein	AT4G19420	+5.45	69%	1 × 10^−^^75^
8874,0		Pectin lyase-like superfamily protein	AT1G11920	+3.10	72%	3 × 10^−^^64^
13011,0		Pectate lyase family protein	AT1G67750	−3.55	77%	0.0
14701,76034		Pectinacetylesterase family protein	AT3G05910	−3.75	73%	1 × 10^−^^101^
14701,45231		Pectinacetylesterase family protein	AT4G19420	−4.87	69%	1 × 10^−^^75^

## Data Availability

The data presented in this study are openly available in NCBI (https://www.ncbi.nlm.nih.gov/geo/, reference number GSE164096.

## Supplementary Materials

The following are available online at https://www.mdpi.com/1422-0067/22/2/882/s1, Figure S1: Effect of *P tracheiphilus* on *C. jambhiri* phenotype, Figure S2: Overview of the number of transcripts and unigenes in different length intervals, Figure S3: Validation of DEGs in *Pt* vs. CK comparison by Real Time qRT-PCR, Figure S4: KOG function classification, Figure S5: Scheme of the metabolic pathways involved in the “Plant hormone” category, Figure S6: Scheme and components of the photosynthetic electron flow including CF0F1-ATP synthase, Table S1: Real-time detection of *P. tracheiphilus* in inoculated plants, Table S2: Primers used to validate the RNAseq experiment by real time PCR.

## References

[1] Talon M., Gmitter F.G. (2008). Citrus genomics. Int. J. Plant Genom..

[2] Ruggieri G. (1949). L’attuale problema del “mal secco” degli Agrumi nelle sue immediate finalità pratiche. Annali Della Sperimentazione Agraria.

[3] EPPO Global Database. https://gd.eppo.int/taxon/DEUTTR.

[4] Russo R., Caruso M., Arlotta C., Lo Piero A.R., Nicolosi E., Di Silvestro S. (2020). Identification of Field Tolerance and Resistance to Mal Secco Disease in a Citrus Germplasm Collection in Sicily. Agronomy.

[5] Nigro F., Ippolito A., Salerno M.G. (2011). Mal secco disease of citrus: A journey through a century of research. J. Plant Pathol..

[6] EFSA Panel Plant Health (2014). Scientific Opinion on the risks to plant health posed by *Phytophthora fragariae* Hickman var. fragariae in the EU territory, with the identification and evaluation of risk reduction options. EFSA J..

[7] Wu G.A., Terol J., Ibanez V., López-García A., Pérez-Román E., Borredá C., Domingo C., Tadeo F.R., Carbonell-Caballero J., Alonso R. (2018). Genomics of the origin and evolution of Citrus. Nature.

[8] Bowman K., Joubert J., Talon M., Caruso M., Gmitter F.G. (2020). Citrus Rootstocks. The Genus Citrus.

[9] Savastano L. (1925). La cura del “mal secco” negli alberi fruttiferi. Bollettino della Stazione Sperimentale di Agrumicoltura e Frutticoltura.

[10] Catara A., Catara V. (2019). Il “mal secco” degli agrumi, da un secolo in Sicilia. Memorie e Rendiconti.

[11] Migheli Q., Cacciola S.O., Balmas V., Pane A., Ezra D., di San Lio G.M. (2009). Mal secco disease caused by *Phoma tracheiphila*: A potential threat to lemon production worldwide. Plant Dis..

[12] Batuman O., Ritenour M., Vicent A., Li H., Hyun J.-W., Catara V., Ma H., Cano L.M., Talon M., Caruso M., Gmitter F.G. (2020). Diseases caused by fungi and oomycetes. The Genus Citrus.

[13] Ruggieri G. (1956). “Mal secco” degli agrumi e attuali mezzi di lotta. Rivista Agrumicoltura.

[14] Nachmias A., Barash I., Solel Z., Strobel G.A. (1977). Translocation of mal secco toxin in lemons and its effect on electrolyte leakage, transpiration, and citrus callus growth. Phytoparasitica.

[15] Fogliano V., Graniti A., Marchese A., Ritieni A., Randazzo G., Visconti A., Kumari S. (1994). Purification of a phytotoxic glycoprotein from the ‘malseccin’ complex produced in culture by *Phoma tracheiphila*. Proceedings of the 9th Congress of the Mediterranean Phytopathological Union.

[16] Fogliano V., Marchese A., Scaloni A., Ritieni A., Visconti A., Randazzo G., Graniti A. (1998). Characterization of a 60 KDa phytotoxic glycoprotein produced by *Phoma tracheiphila* and its relation to malseccin. PMPP.

[17] Fedoroff N. (2006). Redox regulatory mechanisms in cellular stress responses. Ann. Bot..

[18] Reverberi M., Betti C., Fabbri A.A., Zjalic S., Spadoni S., Mattei B., Fanelli C. (2008). A role for oxidative stress in the *Citrus limon/Phoma tracheiphila* interaction. Plant Pathol..

[19] Gentile A., La Malfa S., Farooq M., Pisante M. (2019). New breeding techniques for sustainable agriculture. Innov. Sustain. Agric..

[20] Salonia F., Ciacciulli A., Poles L., Pappalardo H.D., La Malfa S., Licciardello C. (2020). New plant breeding techniques in citrus for the improvement of important agronomic traits. A Review. Front. Plant Sci..

[21] Poles L., Licciardello C., Distefano G., Nicolosi E., Gentile A., La Malfa S.L. (2020). Recent advances of in vitro culture for the application of new breeding techniques in citrus. Plants.

[22] Fan J., Chen C., Yu Q., Brlansky R.H., Lia Z.G., Gmitter F.G. (2011). Comparative iTRAQ proteome and transcriptome analyses of sweet orange infected by “*Candidatus* Liberibacter asiaticus”. Physiol. Plant.

[23] Fan J., Chen C., Yu Q., Khalaf A., Achor D.S., Brlansky R.H., Moore G.A., Li Z.G., Frederick G., Gmitter F.G. (2012). Comparative transcriptional and anatomical analyses of tolerant rough lemon and susceptible sweet orange in response to ‘*Candidatus* Liberibacter asiaticus’ infection. MPMI.

[24] Hu Y., Zhong X., Liu X., Lou B., Zhou C., Wang X. (2017). Comparative transcriptome analysis unveils the tolerance mechanisms of *Citrus hystrix* in response to ‘*Candidatus* Liberibacter *Asiaticus*’ infection. PLoS ONE.

[25] Yu Q., Chen C., Du D., Huang M., Yao J., Yu F., Brlansky R.H., Gmitter F.G. (2017). Reprogramming of a defense signaling pathway in rough lemon and sweet orange is a critical element of the early response to ‘*Candidatus Liberibacter* asiaticus’. Hortic. Res..

[26] Naveed Z.A., Huguet-Tapia J.C., Ali G.S. (2019). Transcriptome profile of Carrizo citrange roots in response to *Phytophthora parasitica* infection. J. Plant Interact..

[27] Arce-Leal A.P., Bautista R., Rodríguez-Negrete E.A., Manzanilla-Ramírez M.A., Velázquez-Monreal J.J., Santos-Cervantes M.E., Méndez-Lozano J., Beuzón C.R., Bejarano E.R., Castillo A.G. (2020). Gene expression profile of Mexican lime (*Citrus aurantifolia*) trees in response to Huanglongbing disease caused by Candidatus Liberibacter asiaticus. Microorganisms.

[28] Licciardello G., Grasso F.M., Bella P., Cirvilleri G., Grimaldi V., Catara V. (2006). Identification and detection of Phoma tracheiphila, causal agent of citrus mal secco disease, by real-time polymerase chain reaction. Plant Dis..

[29] Woodward A.W., Bartel B. (2005). Auxin: Regulation, Action and Interaction. Ann. Bot..

[30] Dempsey D.A., Vlot A.C., Wildermuth M.C., Klessig D.F. (2011). Salicylic acid biosynthesis and metabolism. Arab. Book.

[31] Libault M., Wan J., Czechowski T., Udvardi M., Stacey G. (2007). Identification of 118 *Arabidopsis* transcription factor and 30 ubiquitin-ligase genes responding to chitin, a plant-defense elicitor. MPMI.

[32] Ryu H.S., Han M., Lee S.K., Cho J.I., Ryoo N., Heu S., Lee Y.H., Bhoo S.H., Wang G.L., Hahn T.R. (2006). A comprehensive expression analysis of the WRKY gene superfamily in rice plants during defense response. Plant Cell Rep..

[33] Gao Q.M., Venugopal S., Navarre D., Kachroo A. (2011). Low oleic acid-derived repression of jasmonic acid-inducible defense responses requires the WRKY50 and WRKY51 proteins. Plant Physiol..

[34] Lai Z., Vinod K.M., Zheng Z., Fan B., Chen Z. (2008). Roles of *Arabidopsis* WRKY3 and WRKY4 transcription factors in plant responses to pathogens. BMC Plant Biol..

[35] Li J., Brader G., Palva E.T. (2004). The WRKY70 transcription factor: A node of convergence for jasmonate-mediated and salicylate-mediated signals in plant defense. Plant Cell.

[36] Halter T., Imkampe J., Mazzotta S., Wierzba M., Postel S., Buecherl C., Kiefer C., Stahl M., Chinchilla D., Wang X. (2014). The leucine-rich repeat receptor kinase BIR2 is a negative regulator of BAK1 in plant immunity. Curr. Biol..

[37] Jones J., Dangl J. (2006). The plant immune system. Nature.

[38] Wan J., Zhang X.-C., Neece D., Ramonell K.M., Clough S., Kim S.-Y., Stacey M.G., Stacey G. (2008). A LysM receptor-like kinase plays a critical role in chitin signaling and fungal resistance in *Arabidopsis*. Plant Cell.

[39] Daudi A., Cheng Z., O’Brien J.A., Mammarella N., Khan S., Ausubel F.M., Bolwella G.P. (2012). The apoplastic oxidative burst peroxidase in *Arabidopsis* is a major component of pattern-triggered immunity. Plant Cell.

[40] Barna B., Fodor J., Harrach B.D., Pogány M., Király Z. (2012). The Janus face of reactive oxygen species in resistance and susceptibility of plants to necrotrophic and biotrophic pathogens. Plant Physiol. Biochem..

[41] He M., Ding N.-Z. (2020). Plant unsaturated fatty acids: Multiple roles in stress response. Front Plant Sci..

[42] Puglisi I., Lo Cicero L., Lo Piero A.R. (2013). The glutathione S-transferase gene superfamily: An in silico approach to study the post translational regulation. Biodegradation.

[43] Gullner G., Komives T., Király L., Schröder P. (2018). Glutathione S-transferase enzymes in plant-pathogen interactions. Front. Plant Sci..

[44] Jeong J., Cohu C., Kerkeb L., Pilon M., Connolly E.L., Guerinot M.L. (2008). Chloroplast Fe(III) chelate reductase activity is essential for seedling viability under iron limiting conditions. Proc. Natl. Acad. Sci. USA.

[45] Nafisi M., Fimognari L., Sakuragi Y. (2015). Interplays between the cell wall and phytohormones in interaction between plants and necrotrophic pathogens. Phytochemistry.

[46] Shine M.B., Xiao X., Kachroo P., Kachroo A. (2019). Signaling mechanisms underlying systemic acquired resistance to microbial pathogens. Plant Sci..

[47] Noman A., Aqeel M., Qari S.H., Al Surhanee A.A., Yasin G., Alamri S., Hashem M., Al-Saadi A.M. (2020). Plant hypersensitive response vs pathogen ingression: Death of few gives life to others. Microb. Pathog..

[48] Fu Z.Q., Dong X. (2013). Systemic acquired resistance: Turning local infection into global defense. Annu. Rev. Plant Biol..

[49] Llorente F., Muskett P., Sánchez-Vallet A., López G., Ramos B., Sánchez-Rodríguez C., Jordá L., Parker J., Molina A. (2008). Repression of the auxin response pathway increases *Arabidopsis* susceptibility to necrotrophic fungi. Mol. Plant.

[50] Gentile A., Deng Z., La Malfa S., Distefano G., Domina F., Vitale A., Polizzi G., Lorito M., Tribulato E. (2007). Enhanced resistance to *Phoma tracheiphila* and *Botrytis cinerea* in transgenic lemon plants expressing a *Trichoderma harzianum* chitinase gene. Plant Breed..

[51] Eulgem T., Rushton P.J., Robatzek S., Somssich I.E. (2000). The WRKY superfamily of plant transcription factors. Plant Sci..

[52] Ülker B., Somssich I.E. (2004). WRKY transcription factors: From DNA binding towards biological function. Curr. Opin. Plant Biol..

[53] Zhang Y., Wang L. (2005). The WRKY transcription factor superfamily: Its origin in eukaryotes and expansion in plants. BMC Evol. Biol..

[54] Du L., Chen Z. (2000). Identification of genes encoding receptorlike protein kinases as possible targets of pathogen- and salicylic acid-induced WRKY DNA-binding proteins in *Arabidopsis*. Plant J..

[55] Eulgem T., Rushton P.J., Schmelzer E., Hahlbrock K., Somssich I.E. (1999). Early nuclear events in plant defence signalling: Rapid gene activation by WRKY transcription factors. EMBO.

[56] Turck F., Zhou A., Somssich I.E. (2004). Stimulus-dependent, promoterspecific binding of transcription factor WRKY1 to its native promoter and the defense-related gene PcPR1-1 in Parsley. Plant Cell.

[57] Yu D., Chen C., Chen Z. (2001). Evidence for an important role of WRKY DNA binding proteins in the regulation of NPR1 gene expression. Plant Cell.

[58] Dong J., Chen C., Chen Z. (2003). Expression profiles of the Arabidopsis WRKY gene superfamily during plant defense response. Plant Mol. Biol..

[59] Rouet M.A., Mathieu Y., Barbier-Brygoo H., Laurière C. (2006). Characterization of active oxygen-producing proteins in response to hypo-osmolarity in tobacco and *Arabidopsis* cell suspensions: Identification of a cell wall peroxidase. J. Exp. Bot..

[60] Lherminier J., Elmayan T., Fromentin J., Elaraqui K.T., Vesa S., Morel J., Verrier J.L., Cailleteau B., Blein J.P., Simon-Plas F. (2009). NADPH oxidase-mediated reactive oxygen species production: Subcellular localization and reassessment of its role in plant defense. Mol. Plant Microbe Interact..

[61] Chen Q., Yang G. (2020). Signal function studies of ROS, especially RBOH-dependent ROS, in plant growth, development and environmental stress. J. Plant Growth Regul..

[62] Liao W., Ji L., Wang J., Chen Z., Ye M., Ma H., An X. (2014). Identification of glutathione S-transferase genes responding to pathogen infestation in *Populus tomentosa*. Funct. Integr. Genom..

[63] Berger S., Papadopoulos M., Schreiber U., Kaiser W., Roitsch T. (2004). Complex regulation of gene expression, photosynthesis and sugar levels by pathogen infection in tomato. Physiol. Plant.

[64] Braun H.P. (2020). The Oxidative Phosphorylation system of the mitochondria in plants. Mitochondrion.

[65] Talbot N.J. (2010). Living the sweet life: How does a plant pathogenic fungus acquire sugar from plants?. PLoS Biol..

[66] Barrett H.C., Rhodes A.M. (1976). A numerical taxonomic study of affinity relationships in cultivated citrus and its close relatives. Syst. Bot..

[67] Salerno M., Catara A. (1967). Ricerche sul “mal secco” degli Agrumi (*Deutero-phoma tracheiphila* Petri). IV. Comportamento parassitario del fungo in ospiti diversi dagli Agrumi. Tecnica Agricola.

[68] Springer N.M. (2010). Isolation of plant DNA for PCR and genotyping using organic extraction and CTAB. Cold Spring Harb. Protoc..

[69] Sicilia A., Testa G., Santoro D.F., Cosentino S.L., Lo Piero A.R. (2019). RNASeq analysis of giant cane reveals the leaf transcriptome dynamics under long-term salt stress. BMC Plant Biol..

[70] Benjamini Y., Hochberg Y. (1995). Controlling the false discovery rate: A practical and powerful approach to multiple testing. J. R. Stat. Soc. Ser. B-Methodol..

