# Supplementary material for: De Novo Transcriptome Sequencing of Rough Lemon Leaves (Citrus jambhiri Lush.) in Response to Plenodomus tracheiphilus Infection
Source: Int J Mol Sci. 2021 Jan 17;22(2):882. doi: 10.3390/ijms22020882 (PMC7830309; doi:10.3390/ijms22020882)
Supplement: Supplementary file 1 [file ijms-22-00882-s001.zip › Supplementary files/Figure S1.docx]

**Figure S1 - Effect of *P tracheiphilus* on *C. jambhiri* phenotype. A)** Inoculation site showing the typical MDS symptoms. **B)** Picture of the plants after 15 days from inoculation. On the top, control plants of rough lemon on good healthy state; On the bottom, inoculated plants of rough lemon that showed typical symptoms of MSD and micronutrients deficiency.

A)


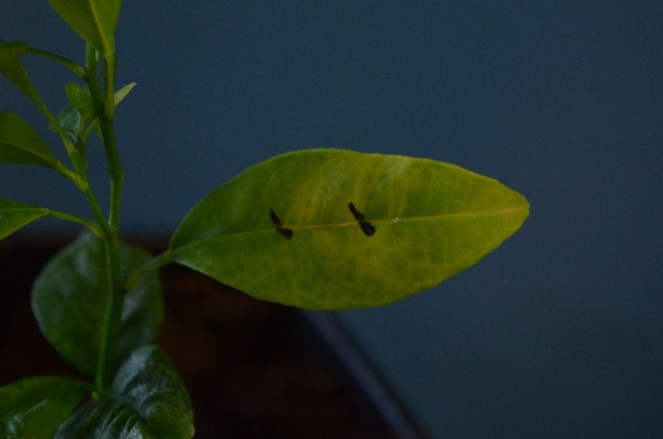


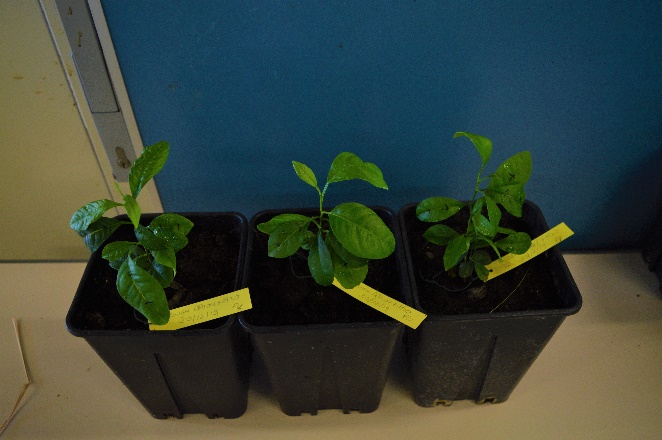

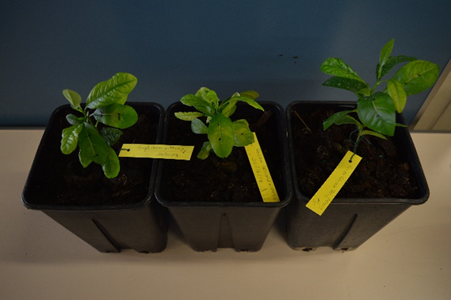
B)
