# Supplementary material for: De Novo Transcriptome Sequencing of Rough Lemon Leaves (Citrus jambhiri Lush.) in Response to Plenodomus tracheiphilus Infection
Source: Int J Mol Sci. 2021 Jan 17;22(2):882. doi: 10.3390/ijms22020882 (PMC7830309; doi:10.3390/ijms22020882)
Supplement: Supplementary file 1 [file ijms-22-00882-s001.zip › Supplementary files/Figure S3.docx]

| **Cluster ID** | **Log_2_ Fold change Illumina** | **Log_2_ Fold change rt-PCR** |
| --- | --- | --- |
| Cluster-7300.1 | 3.13 | 2.16 |
| Cluster-20465.1 | 3.09 | 2.11 |
| Cluster-14701.26429 | 8.96 | 7.35 |
| Cluster-14701.59152 | 5.28 | 4.07 |
| Cluster-14701.49196 | 4.1 | 4.11 |
| Cluster-14701.30701 | -4.15 | -0.26 |
| Cluster-17016.0 | -2.3 | -1.87 |
| Cluster-14701.18090 | -4.24 | -1.18 |
| Cluster-14701.83847 | -2.45 | -0.60 |
| Cluster-14701.23987 | -3.58 | -2.12 |


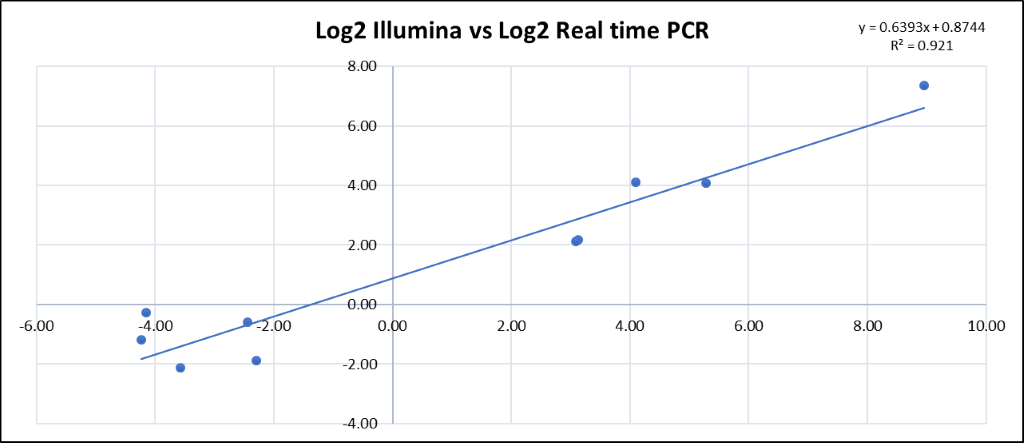


Figure S3 - Validation of DEGs in *Pt* vs CK comparison by Real Time qRT-PCR
