# Supplementary material for: De Novo Transcriptome Sequencing of Rough Lemon Leaves (Citrus jambhiri Lush.) in Response to Plenodomus tracheiphilus Infection
Source: Int J Mol Sci. 2021 Jan 17;22(2):882. doi: 10.3390/ijms22020882 (PMC7830309; doi:10.3390/ijms22020882)
Supplement: Supplementary file 1 [file ijms-22-00882-s001.zip › Supplementary files/Figure S5.docx]

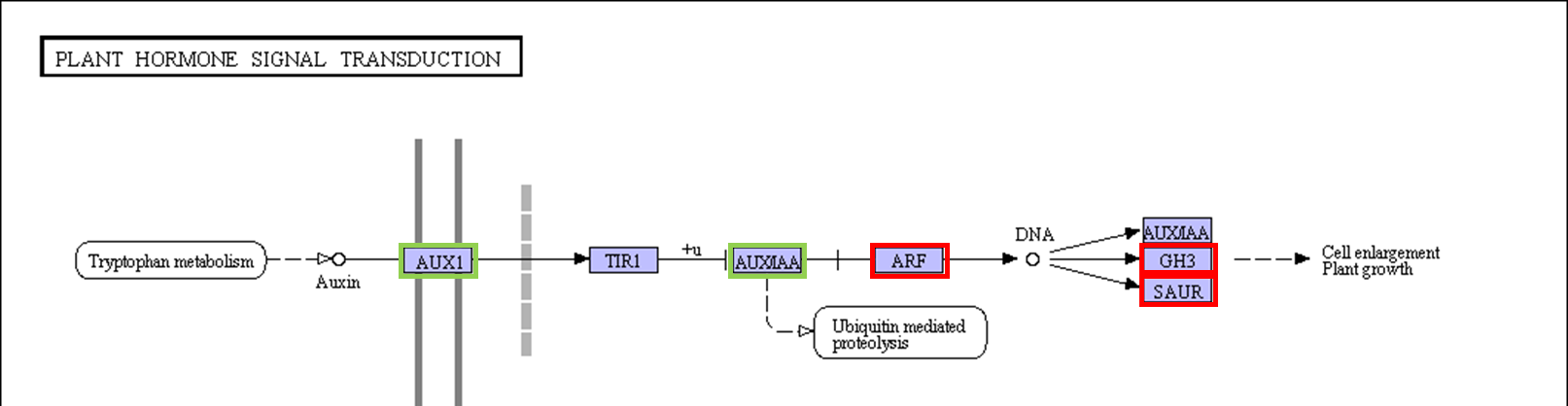


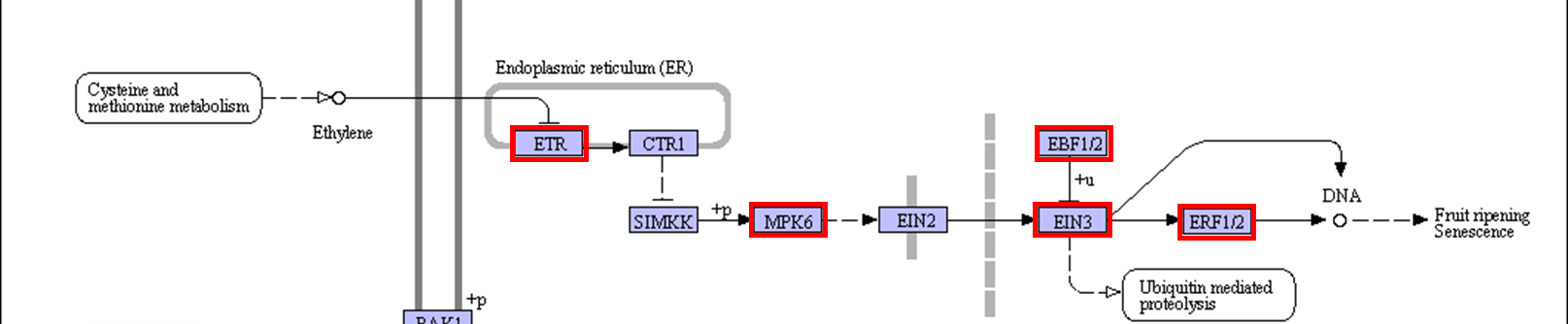


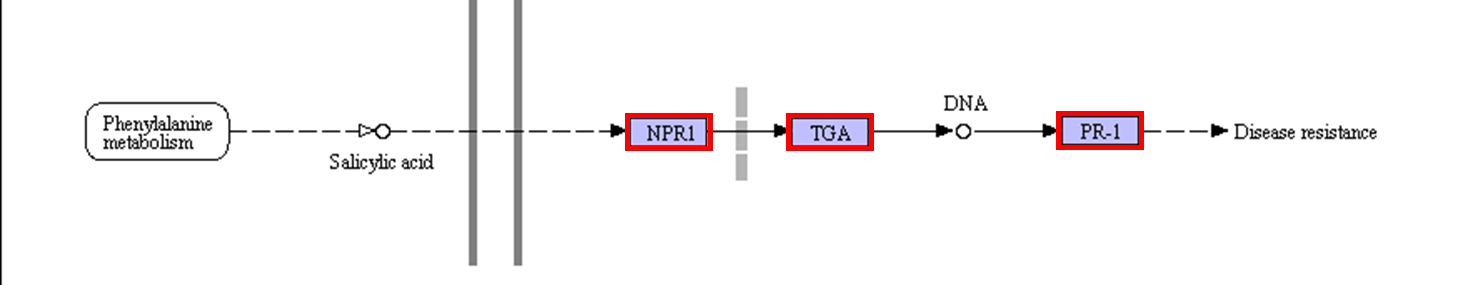


Figure S5 – Scheme of the metabolic pathways involved in the “Plant hormone” category (clusters in boxes surrounded by a green line are down regulated, clusters in boxes surrounded by a red line are up- regulated)
