# Supplementary material for: De Novo Transcriptome Sequencing of Rough Lemon Leaves (Citrus jambhiri Lush.) in Response to Plenodomus tracheiphilus Infection
Source: Int J Mol Sci. 2021 Jan 17;22(2):882. doi: 10.3390/ijms22020882 (PMC7830309; doi:10.3390/ijms22020882)
Supplement: Supplementary file 1 [file ijms-22-00882-s001.zip › Supplementary files/Table S1.docx]

| **Pattern** | **Cluster ID** | **Annotation** | **Primer F** | **Primer R** |
| --- | --- | --- | --- | --- |
| *Pt*_vs_CK /Up | 7300.1 | Peroxisomal membrane protein PMP22 | TGCCATTTTCAAGGGAAGGGAC | TTCTCGGAATTGCTGAGGGACG |
| *Pt*_vs_CK /Up | 20465.1 | LRR receptor-like serine/threonine-protein kinase BIR2 | TGCCAGCCTCCATTTCTGCATC | GCACAACCCCGATTCCATACAC |
| *Pt*_vs_CK /Up | 14701.26429 | Pathogenesis-related protein 1-like | GCGACTGCAATCTTGTGCATTC | TATAGTGCCCACACACCTTGCC |
| *Pt*_vs_CK /Up | 14701.59152 | Chitin elicitor receptor kinase 1 | GCCGAGACATACTATGCCAACC | TCAGGGATGAAAACCAAGCCAC |
| *Pt*_vs_CK /Up | 14701.49196 | Salicylic acid-binding protein 2 | TGCACACATTCCAGGCATACAG | GCCGTCCTCTTTTCCCATCTTC |
| *Pt*_vs_CK /Down | 14701.30701 | Calcium-transporting ATPase 4, plasma membrane-type | AGCTCTGAGAACTCTCTGCCTG | TTTGAACCGCCTCCTTGACTCC |
| *Pt*_vs_CK /Down | 17016.0 | Vegetative cell wall protein gp1 | ATGATGAGTTGCCCCAACAAGG | TCCGTATGACGGATAAGCGGAG |
| *Pt*_vs_CK /Down | 14701.18090 | Pectinesterase 2 | ACTCCAAACACTTCGCCGTC | AGCAATCAGCCCATGCAACC |
| *Pt*_vs_CK /Down | 14701.83847 | Calcium-dependent protein kinase 17 | GGCCAGAATTTGCATGGGACAG | CAAGATAACGCCAGCACTCCAC |
| *Pt*_vs_CK /Down | 14701.23987 | CKI1_ARATH Histidine kinase CKI1 | AGTTCGCGCAGCAGTAAAGAAG | AATCCCCTTGCCCGTATCATCC |
| All | 14701.53432 | Actin | CTCACTGAAGCACCACTCAACC | CACCATCTCCAGAGTCAAGCAC |

Table S1 – Primers used to validate the RNAseq experiment by real time PCR
