# Supplementary material for: De Novo Transcriptome Sequencing of Rough Lemon Leaves (Citrus jambhiri Lush.) in Response to Plenodomus tracheiphilus Infection
Source: Int J Mol Sci. 2021 Jan 17;22(2):882. doi: 10.3390/ijms22020882 (PMC7830309; doi:10.3390/ijms22020882)
Supplement: Supplementary file 1 [file ijms-22-00882-s001.zip › Supplementary files/Table S2.docx]

| **Sample Name** | **Ct value** | **Sandard deviation Ct value** | **Quantification** |
| --- | --- | --- | --- |
| NTC | Undetermined |  |  |
| NTC | Undetermined |  |  |
| T1 | 24.758 | 0.436 | 0.074882 |
| T1 | 24.1411 | 0.436 | 0.107628 |
| T2 | 22.7171 | 0.46 | 0.248684 |
| T2 | 23.3674 | 0.46 | 0.169646 |
| T3 | 23.1696 | 0.0701 | 0.190574 |
| T3 | 23.0705 | 0.0701 | 0.202013 |
| C1 | Undetermined |  |  |
| C1 | Undetermined |  |  |
| C2 | Undetermined |  |  |
| C2 | Undetermined |  |  |
| C3 | Undetermined |  |  |
| C3 | Undetermined |  |  |

**Table S2 - Real-time detection of *P. tracheiphilus* in inoculated plants.**

Ct (threshold cycle), T (treated plant), C (control plant), NTC (negative control, plants inoculated with water).
