# Supplementary figures and images for: De Novo Transcriptome Sequencing of Rough Lemon Leaves (Citrus jambhiri Lush.) in Response to Plenodomus tracheiphilus Infection
Source: Int J Mol Sci. 2021 Jan 17;22(2):882. doi: 10.3390/ijms22020882 (PMC7830309; doi:10.3390/ijms22020882)

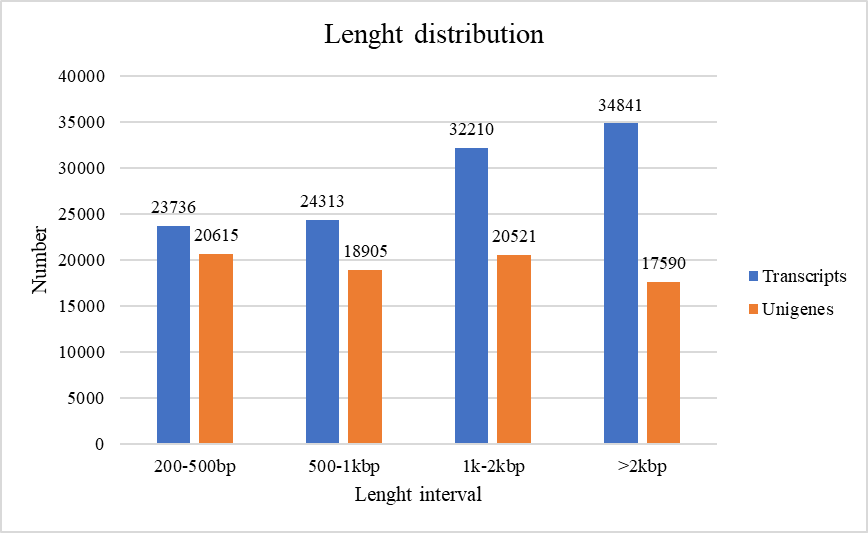


Figure S2 - Overview of the number of transcripts and unigenes in different length intervals.

Supplement: Supplementary file 1 [file ijms-22-00882-s001.zip › Supplementary files/Figure S2.docx]

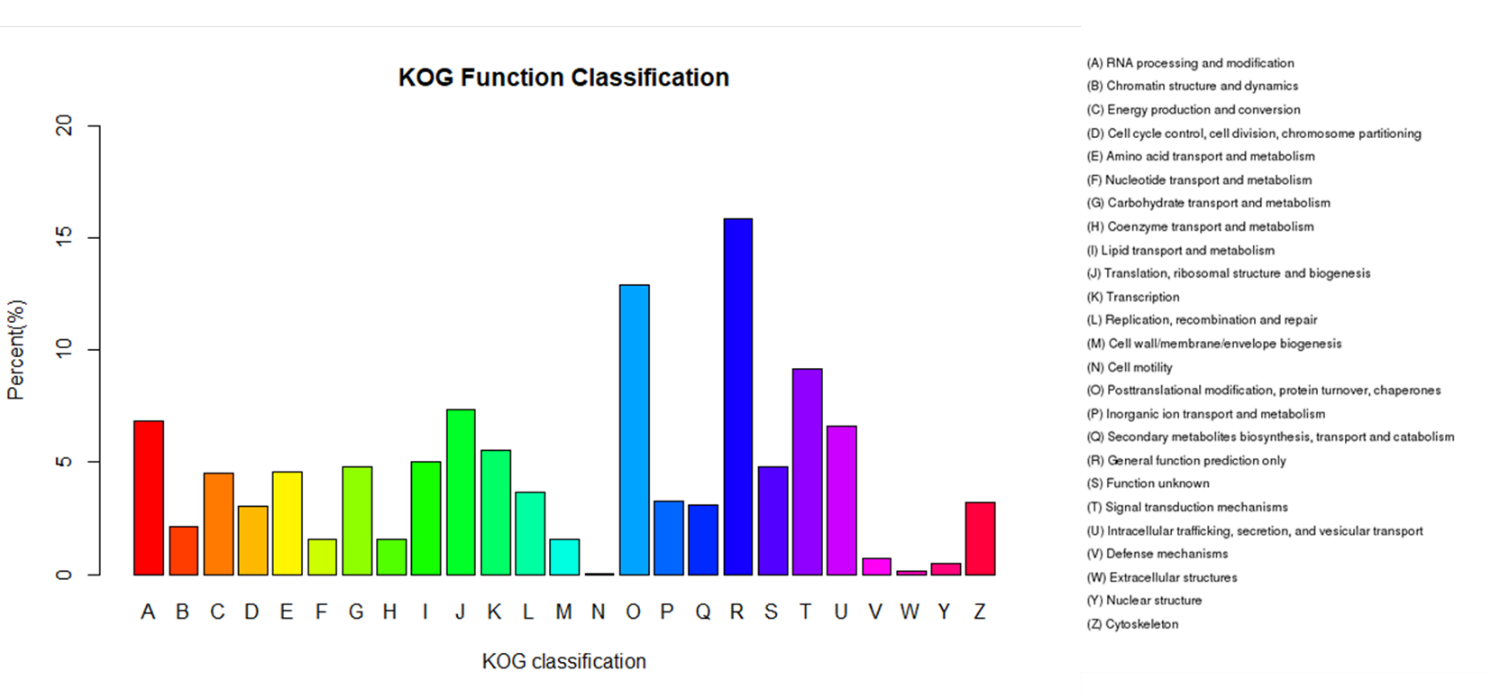


Figure S4 – KOG function classification

Supplement: Supplementary file 1 [file ijms-22-00882-s001.zip › Supplementary files/Figure S4.docx]
